# Mapping the genomic architecture of adaptive traits with interspecific introgressive origin: a coalescent-based approach

**DOI:** 10.1186/s12864-015-2298-2

**Published:** 2016-01-11

**Authors:** Hussein A. Hejase, Kevin J. Liu

**Affiliations:** Department of Computer Science and Engineering, Michigan State University, 428 S. Shaw Lane, East Lansing, 48824 MI USA

**Keywords:** Introgression, Gene flow, Incomplete lineage sorting, Association mapping, Population structure, Phylogenomic, Coalescent, Mouse

## Abstract

Recent studies of eukaryotes including human and Neandertal, mice, and butterflies have highlighted the major role that interspecific introgression has played in adaptive trait evolution. A common question arises in each case: what is the genomic architecture of the introgressed traits? One common approach that can be used to address this question is association mapping, which looks for genotypic markers that have significant statistical association with a trait. It is well understood that sample relatedness can be a confounding factor in association mapping studies if not properly accounted for. Introgression and other evolutionary processes (e.g., incomplete lineage sorting) typically introduce variation among local genealogies, which can also differ from global sample structure measured across all genomic loci. In contrast, state-of-the-art association mapping methods assume fixed sample relatedness across the genome, which can lead to spurious inference. We therefore propose a new association mapping method called Coal-Map, which uses coalescent-based models to capture local genealogical variation alongside global sample structure. Using simulated and empirical data reflecting a range of evolutionary scenarios, we compare the performance of Coal-Map against EIGENSTRAT, a leading association mapping method in terms of its popularity, power, and type I error control. Our empirical data makes use of hundreds of mouse genomes for which adaptive interspecific introgression has recently been described. We found that Coal-Map’s performance is comparable or better than EIGENSTRAT in terms of statistical power and false positive rate. Coal-Map’s performance advantage was greatest on model conditions that most closely resembled empirically observed scenarios of adaptive introgression. These conditions had: (1) causal SNPs contained in one or a few introgressed genomic loci and (2) varying rates of gene flow – from high rates to very low rates where incomplete lineage sorting dominated as a primary cause of local genealogical variation.

**Electronic supplementary material:**

The online version of this article (doi:10.1186/s12864-015-2298-2) contains supplementary material, which is available to authorized users.

## Background

**Fig. 1:**
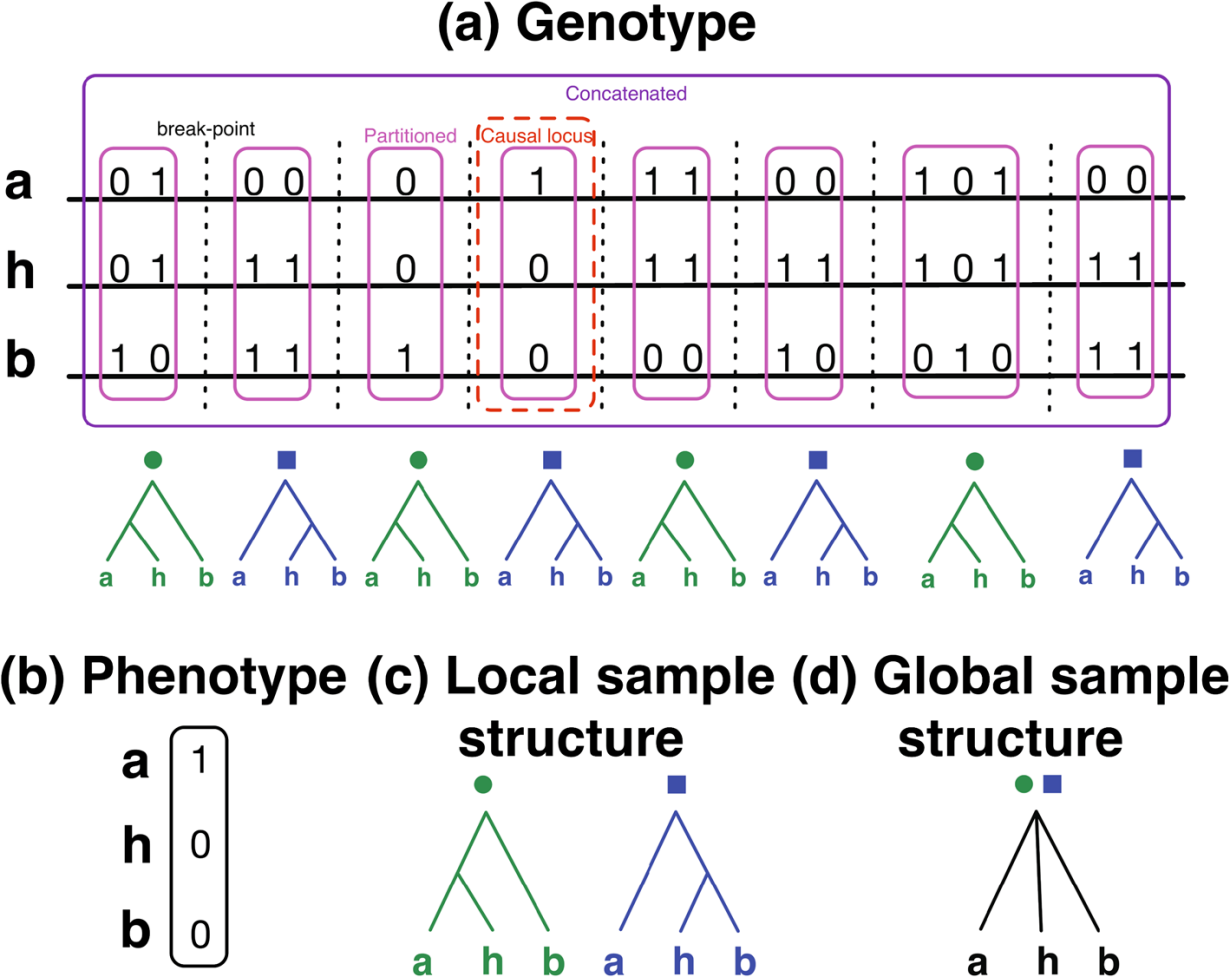
Local genealogical variation: a sequence level view. The illustration shows an example outcome of evolution under the species phylogeny depicted in Fig. 2, where individual *a* is sampled from population A, individual *b* is sampled from population B, and individual *h* is sampled from population H. **a** A haploid genome sequence is shown for each of the three individuals. Different genealogies are observed for different genomic loci, depending on the specific coalescent history of each locus. Each locus is colored green or blue based on the topology of the genealogy at that locus. The ancestral and derived alleles are represented as 0 and 1, respectively. In our example, the locus marked with a dashed red box contains a causal SNP that contributes to the observed phenotype shown in (**b**). **c** Sample structure (or the evolutionary relationships between samples) in green loci differs from sample structure in blue loci. **d** In our example, global sample structure (i.e., sample structure measured across all sites) takes the form of a star tree. Notice that global sample structure differs from local sample structure in any single locus

**Fig. 2:**
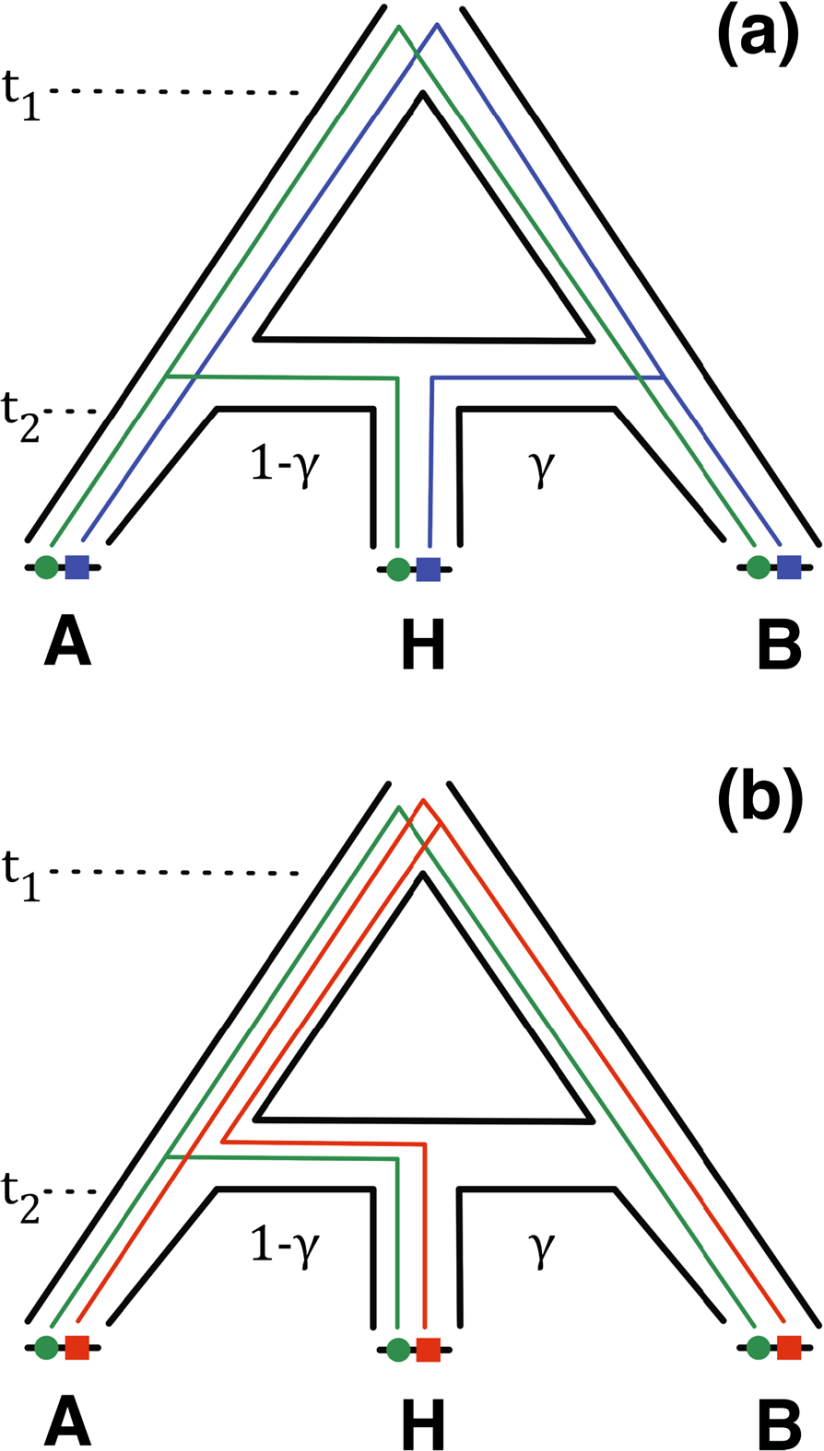
Local genealogical variation: a species phylogeny view. The illustration shows two different pairs of incongruent local genealogies evolving within a species phylogeny: one pair involving incomplete lineage sorting, and the other involving hybrid origin from two different parental populations. The species phylogeny involves three populations A, B, and H. Populations A and B diverged at time *t*_1_. At time *t*_2_, a hybridization event between the ancestral populations of A and B occurred, giving rise to a hybrid population H. **a** The genealogies of two different loci (green and blue) are shown. A lineage in H originated from the ancestral population of B with probability *γ* (blue locus) or the ancestral population of A with probability 1−*γ* (green locus). **b** The genealogies of two different loci (green and red) are shown. The H alleles at both loci originated from the ancestral population of A. For the green locus, the H lineage and A lineage coalesce between time *t*_2_ and *t*_1_. For the red locus, tracing backwards in time we find that no coalescence events occur until after time *t*_1_, resulting in ancestral polymorphism and incomplete lineage sorting. Note that local genealogical variation can involve both topological differences (as shown here) and branch length differences

Adaptive interspecific introgression has played a key role in the evolution of novel traits in many eukaryotic organisms. Examples include hemoglobin concentration in Tibetans as an adaptation to high-altitude environments [1, 2], mimetic butterfly wing patterns [3], and pesticide resistance in house mice [4, 5]. Figure 1 illustrates the effects of adaptive introgression at the genomic sequence level. The phylogeny of three present-day species A, B, and H is shown in Fig. 2. Species A and B diverged from a most recent common ancestor at time *t*_1_. At time *t*_2_, a new hybrid population was formed by hybridization between species A and B. We assume that ploidy is preserved across the phylogeny. In a first generation hybrid offspring, half of the alleles in its genome are expected to come from a parent individual in species A and the other half from a parent individual in species B. A locus in a first-generation hybrid genome has ancestry either from species A or from species B, depending upon the outcome of genetic recombination. We refer to a contiguous genomic subsequence with ancestry from a single parental species as a tract. The evolutionary history of a tract in a first-generation hybrid is represented by either the blue or the green genealogy shown in Fig. 1. Over subsequent generations, back-crossing, recombination, and incomplete lineage sorting of unlinked loci cause fragmentation of genomic tracts present in the first generation of hybrids. The net result is introgression, where genetic material from one of the parental species is incorporated into species H. Natural selection and resulting genetic hitchhiking effects further influence the outcome of introgression. Negative selection fragments and removes introgressed tracts containing maladaptive alleles; on the other hand, positive selection can maintain introgressed tracts containing adaptive alleles over time. Adaptive introgression therefore has two major effects on evolutionary relatedness: (1) local genealogies can vary substantially across different genomic loci, and (2) local genealogical variation resembles a mosaic where tracts in present-day genomes depend upon the outcome of all of these evolutionary processes acting in combination. Longer tracts reflect either more recent hybridization events or adaptive introgression. New computational methodologies have been proposed to perform phylogenomic inference under models that explicitly incorporate the complex interplay of these different evolutionary processes [6–11]. Of particular relevance is PhyloNet-HMM [11], a method that we collaboratively developed. PhyloNet-HMM is the first method capable of scalable phylogenomic inference on three or more genomes under a model that includes these evolutionary processes.

**Fig. 3:**
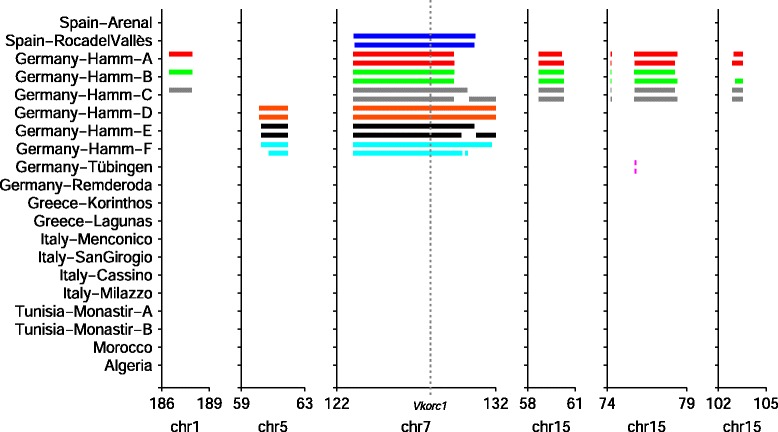
Genomic tracts in *Mus musculus* samples that were inferred to originate via introgression with *M. spretus*. PhyloNet-HMM [11] was used to infer genomic signatures of interspecific introgression using the procedure described by Liu et al. [5]. Only western European and north African samples are shown. Samples are labeled by country, city/region (if multiple samples originated from the same country), and identifier (if multiple samples originated from the same city/region). The haploid chromosomes are shown for each sample in a separate track and colored a non-white color to denote introgressive origin or white otherwise; each sample is assigned a different color for visibility. The chart shows a subset of the introgressed genomic regions that were inferred to originate due to the recent selective sweep associated with anticoagulant pesticide use. The location of the *Vkorc1* gene is highlighted, which is known to contribute to anticoagulant resistance in both mice and humans [4, 12]. Panel adapted from [5]

Recently in collaboration with others, we used PhyloNet-HMM to uncover genome-wide signatures of introgression between natural populations of *Mus musculus* and its sister species *Mus spretus* [5]. Dozens of introgressed genomic tracts were more than a megabase in length and inferred to be recent in origin due to the introduction of rodenticide use in and outside of the regions of sympatry between the two species. The longest of these genomic tracts – around 10 Mb in length – harbored mutations in the *Vkorc1* gene which are known to contribute to resistance and susceptibility to warfarin [4, 12], a widely used anticoagulant rodenticide (Fig. 3). Other introgressed tracts are suspected to harbor other adaptive alleles. The study also uncovered introgressed tracts that were putatively more ancient in origin and which had unknown functional roles. Several major open questions follow from this work: (1) What other introgressed alleles played a causative role in the evolution of the rodenticide resistance trait? (2) What other traits were involved in introgression between the mouse species, and which introgressed alleles contributed to the evolution of these traits?

Genome-wide association (GWA) mapping methods can be used to obtain important clues concerning these questions. GWA methods are widely used throughout the life sciences to investigate the genetic architecture of complex traits, particularly in natural human populations and laboratory strains of model organisms [13]. The goal of GWA mapping is to detect significant statistical associations between genomic markers and a trait of interest. An important consideration is that relatedness between sampled individuals, or sample structure, can induce spurious associations between genotypic and phenotypic characters when not properly accounted for [14]. Intuitively, genotypic and phenotypic characters evolved down a common phylogeny (or evolutionary history), introducing covariance that is distinct from covariance due to a causal relationship between a genotypic marker and trait. Depending on study design, sample structure can encompass multiple levels of relatedness. In the case of GWA studies in humans, sample structure can be due to more distant relationships from population subdivision [15], as well as less distant relationships (e.g., family relationships) [16]. In the case of GWA studies of laboratory organisms such as inbred mouse strains, sample structure can include cryptic relatedness due to their artificial origins, which typically involve a complicated breeding history [17].

The statistical methodologies used in GWA studies can also be used to investigate the genomic architecture of traits involved in adaptive introgression between different species. However, the sample structure is typically more complex than in human populations and artificial laboratory organisms such as classical and wild-derived mouse strains. Two contributing factors are: (1) greater evolutionary divergence involving heterogeneous evolutionary processes such as gene flow, recombination, lineage sorting, and natural selection, and (2) genealogical histories of different loci within genomes can vary significantly not only in terms of branch lengths but also topologies. The latter can cause sample relatedness in one locus to differ from other loci as well as the genome as a whole.

Exactly how to account for sample relatedness remains a subject of major debate. The most widely used GWA methods adopt a range of approaches, broadly categorized in terms of their complexity. On one end of the spectrum, a genomic control approach involves computing an inflation factor based on the degree of sample relatedness, which is then used to correct association statistics [18]. On the other end of the spectrum are more highly parameterized fixed-effect models and mixed models where population structure is modeled using either fixed effects or random effects, respectively (reviewed in [19]). Methods based on fixed-effect models and mixed models represent the current state of the art in terms of computational efficiency and accuracy [20, 21]. Among the most accurate and efficient of these methods are EIGENSTRAT [22], EMMA [17] and its successor method EMMAX [21], and GEMMA [23]. Notably, these methods were at the center of a recent debate on exactly how to model sample structure [19, 24, 25]. Only a few existing methods explicitly adopt an evolutionary model in the form of a phylogeny. These include EMMA when used with a kinship matrix based on a Brownian motion model of phenotypic evolution on a phylogenetic tree [17] and PHYLOSTRAT [20], which uses a regression-based approach with a model that includes the bipartitions of a phylogenetic tree computed from genotypic data. Significantly, none of these methods utilize models that model variation in sample relatedness across genomes nor explicitly capture non-tree-like evolution, as in the case of species whose evolution involved adaptive introgression.

More recently, GWA studies have begun to examine admixed human populations (reviewed by [26]), introducing sample structure with greater complexity due to the non-tree-like evolutionary histories resulting from admixture. Shriner et al. [27] and Pasaniuc et al. [28] introduced BMIX and MIXSCORE, respectively, which are tests to simultaneously detect local signatures of admixture and genotypic/phenotypic association. Of the two, BMIX was shown to offer more statistical power and type I error control [27]. Shriner et al. utilized BMIX to perform association mapping on African-American populations and found several new markers that were significantly associated with fasting plasma glucose, coronary heart disease, type 2 diabetes, and breast cancer. Two aspects of the methodological design of BMIX are most relevant to our work. First, a key step in BMIX is local ancestry inference using LAMPANC [29], where each allele at a locus in an admixed genome is inferred to originate from one of two parental populations (CEU and YRI populations in the HapMap study [30]). Importantly, the inference makes use of the assumption that the parental populations are unrelated. A simulation study was performed to evaluate statistical power, which similarly used a model with unrelated parental populations. Second, an intermediate stage of the BMIX algorithm consists of stratified regression of a genotypic character corresponding to a test marker with a phenotypic character. The stratified regression technique adjusts for both local sample structure at the locus as well as global sample structure (i.e., sample structure measured across the entire genomic sequence). Our study proposes an alternative method for association mapping in the context of local variation in sample structure. We note that any such method can be used in place of their stratified regression technique (steps 3 through 5 in the algorithm shown in [27]). Other association mapping methods have been shown to be more accurate than stratified regression. These include the fixed effects model methods and mixed model methods that we consider in our study.

As suggested by [17], the true genealogical histories of the genomic loci in a GWA study could theoretically be used to construct a concise and interpretable model of complex sample structure. The genealogical histories would be especially useful where local genealogies exhibit both topological and branch length variation as is often the case in genomes with adaptively introgressed origins. In practice, local genealogical histories must be inferred. Admixture mapping methods such as LAMPANC address the specific case of inferring local genealogical variation due to genetic admixture (reviewed by [26]). In the context of interspecific introgression involving eukaryotic genomes, additional evolutionary processes have first-order effects on local genealogical variation, including incomplete lineage sorting [31]. New methods based upon the coalescent model [32] and its extensions enable efficient and accurate inference of species phylogenies and local genealogies under models that account for all of the above evolutionary processes [6, 11]. Evolutionary models provide an ideal means to understand the impact of complex sample structure on state-of-the-art association mapping methods. These evolutionary models can be utilized to account for complex sample structure in an association mapping study. We therefore introduce Coal-Map, a new association mapping method. Coal-Map is a computational pipeline which adopts an evolutionary perspective by modeling local genealogical histories in a mixed model association mapping framework. We study the performance of Coal-Map using both synthetic data and empirical data sampled from natural populations of house mouse. Coal-Map improves upon the state-of-the-art in terms of both statistical power and false positive rate.

## Results

### Performance study using simulated genotypic and phenotypic data

**Fig. 4:**
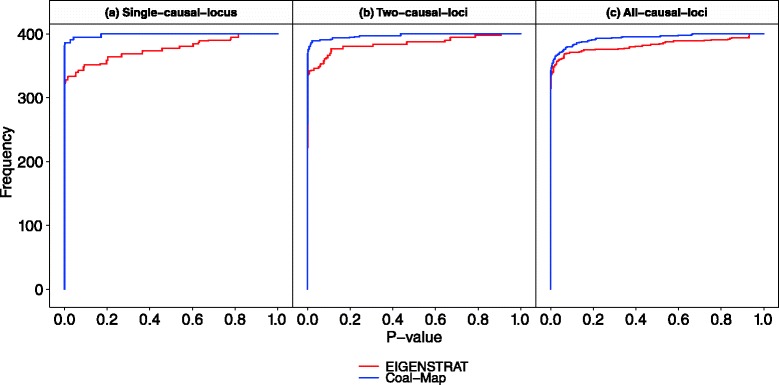
Compared to EIGENSTRAT, Coal-Map has comparable or better power and false positive rate on model conditions involving adaptive gene flow (hybridization frequency *γ*=0.5 and selection coefficient *s*=0.56). True positive rate and false positive rate are shown for both methods using receiver operating characteristic (ROC) curves. **a** Results are shown for the model condition where causal SNPs are drawn from a single locus. Coal-Map and EIGENSTRAT have AUROC of 0.947 and 0.889, respectively. **b** Results are shown for the two-causal-loci model condition. Coal-Map has an AUROC of 0.897 and EIGENSTRAT has an AUROC of 0.859. **c** Results are shown for the all-causal-loci model condition, where Coal-Map and EIGENSTRAT have AUROC of 0.845 and 0.816, respectively

The simulation study included model conditions with adaptive gene flow. A range of genetic architectures were simulated, where one, two, or all loci contained causal markers. Figure 4 compares the performance of Coal-Map and EIGENSTRAT using receiver operating characteristic (ROC) curves on model conditions with the highest level of gene flow (*γ*=0.5). Coal-Map offered better power than EIGENSTRAT across the different model conditions. The performance improvement was significant in terms of area under ROC curve (AUROC) for all model conditions (Delong et al. test [33], *α*=0.05), with *P* values of 2∗10^−14^, 5∗10^−5^, and.006 for the single-causal-locus, two-causal-loci, and all-causal-loci model conditions, respectively. As measured by AUROC, Coal-Map’s performance advantage over EIGENSTRAT was largest on the single-causal-locus model condition (0.947 versus 0.889, respectively) and smaller as more loci contributed causal SNPs. This can also be seen based on each method’s power at typical false positive rates. At a false positive rate (FPR) of 0.05, Coal-Map’s TPR improved upon EIGENSTRAT’s by 0.152 and 0.054 on the single-causal-locus and two-causal-loci model conditions, respectively; on the all-causal-loci model condition, the TPR difference between the two methods was less than 0.039.

**Fig. 5:**
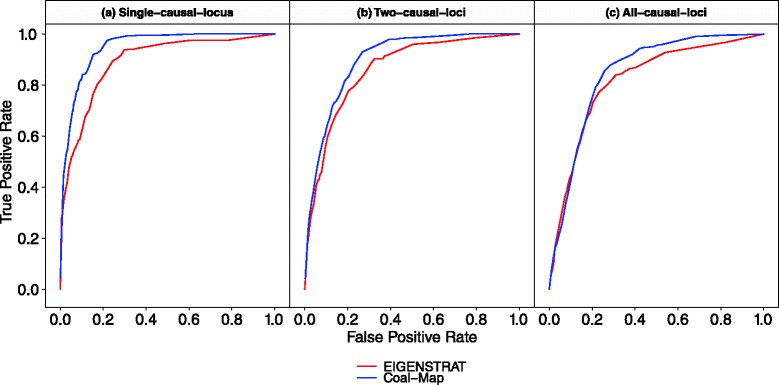
Compared to EIGENSTRAT, Coal-Map has comparable or better power and false positive rate on model conditions involving neutral gene flow (hybridization frequency *γ*=0.5). True positive rate and false positive rate are shown for both methods using receiver operating characteristic (ROC) curves. **a** Results are shown for the model condition where causal SNPs are drawn from a single locus. Coal-Map and EIGENSTRAT have area-under-ROC-curve (AUROC) of 0.938 and 0.870, respectively. **b** Results are shown for the two-causal-loci model condition. Coal-Map has an AUROC of 0.898 and EIGENSTRAT has an AUROC of 0.860. **c** Results are shown for the all-causal-loci model condition, where Coal-Map and EIGENSTRAT have AUROC of 0.837 and 0.827, respectively

**Fig. 6:**
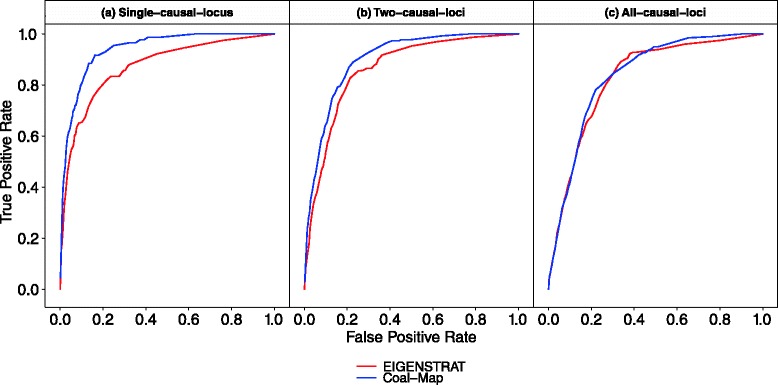
The cumulative histogram of *p*-values reported by Coal-Map and EIGENSTRAT at causal SNPs is shown on model conditions involving neutral gene flow (hybridization frequency *γ*=0.5). Results are shown for the **a** single-causal-locus, **b** two-causal-loci, and **c** all-causal-loci model conditions, respectively. Cumulative frequency is reported over all replicates from a model condition

Coal-Map’s performance advantage over EIGENSTRAT was similarly observed in model conditions that involved a range of hybridization frequencies from *γ*=0.01 to 0.5 and intra-locus linkage that emulated selective sweep effects (but did not directly incorporate positive selection). The synthetic traits incorporated genetic contributions from one, two, or all loci. In Fig. 5, the performance of Coal-Map and EIGENSTRAT on model conditions with the highest level of gene flow (*γ*=0.5) is shown using ROC curves. Across the different trait architectures (i.e., causal SNPs drawn from one, two, or all loci in the single-causal-locus, two-causal-loci, and all-causal-loci model conditions, respectively), Coal-Map offered comparable or better power than EIGENSTRAT for a given false positive rate. The performance improvement was significant in terms of area under ROC curve (AUROC) for the single-causal-locus and two-causal-loci model conditions but not for the all-causal-loci model conditions (Delong et al. test [33], *α*=0.05). Coal-Map’s performance advantage over EIGENSTRAT was largest on the single-causal-locus model condition: the methods had AUROC values of 0.938 versus 0.870, respectively, and Coal-Map’s TPR improved upon EIGENSTRAT by 0.111 at an FPR of 0.05. As more loci contributed causal SNPs, Coal-Map’s performance advantage was smaller. Overall, Coal-Map reported smaller *p*-values at causal SNPs compared to EIGENSTRAT (Fig. 6).

As we examined single-causal-locus and two-causal-loci model conditions with smaller levels of gene flow, we observed that Coal-Map’s performance advantage over EIGENSTRAT did not diminish and in fact remained roughly the same. As shown in Table 1, the performance improvement of Coal-Map over EIGENSTRAT remained significant as we decreased the gene flow parameter *γ* from 0.5 to 0.01 (Delong et al. test [33], *α*=0.05). Thus, on the model condition with negligible gene flow (i.e., *γ*=0.01), virtually all local genealogical variation was due to incomplete lineage sorting. At an FPR of 0.05, Coal-Map’s TPR improved upon EIGENSTRAT by 0.211, 0.171, and 0.242 on single-causal-locus model conditions with *γ* settings of 0.25, 0.1, and 0.01, respectively; on two-causal-loci model conditions, the corresponding TPR improvements were 0.036, 0.032, and 0.094, respectively. The performance of the two methods on the all-causal-loci model conditions was similar regardless of the amount of gene flow, as measured by either AUROC or TPR at a typical FPR level.

**Table 1:** The performance of Coal-Map and EIGENSTRAT based on area under receiver operating characteristic curve (AUROC) is compared across model conditions involving neutral evolution with incomplete lineage sorting and a wide range of gene flow

	Single-causal-locus		
Hybridization			Corrected
frequency *γ*	Coal-Map	EIGENSTRAT	q value
0.5	0.938	0.870	<10^−5^
0.25	0.935	0.882	<10^−5^
0.1	0.928	0.890	<10^−5^
0.01	0.917	0.845	<10^−5^
	Two-causal-loci		
0.5	0.898	0.860	<10^−5^
0.25	0.911	0.860	<10^−5^
0.1	0.881	0.843	<10^−5^
0.01	0.879	0.834	<10^−5^
	All-causal-loci		
0.5	0.836	0.826	0.16
0.25	0.842	0.808	0.001
0.1	0.854	0.842	0.093
0.01	0.847	0.817	0.002

On single-causal-locus and two-causal-loci model conditions, Coal-Map has AUROC that is significantly better than EIGENSTRAT (Delong et al. test [33] with Benjamini-Hochberg correction [65]; setwise *α*=0.05; *n*=20 for each test) across different hybridization frequencies ranging from a relatively large level of gene flow (*γ*=0.5) to negligible amounts of gene flow (*γ*=0.01). On all-causal-loci model conditions, Coal-Map had a diminished performance advantage in terms of AUROC, and the improvement was either weakly significant or not significant (under the same test)

We explored the sensitivity of Coal-Map to the number of covariates (five and twenty) used to represent the sample structure. We found that Coal-Map’s performance was robust to the number of covariates used to represent sample structure (Additional file 1: Figure S1 and S2). We also explored a trait model with only a genotypic component (i.e., lacking a random effect due to environment). We found that Coal-Map’s performance advantage over EIGENSTRAT was greater than on the model conditions that included both genotypic and environmental effects (Additional file 1: Figure S4). Finally, we observed that modeling local sample structure alone resulted in a marked decrease in performance (Additional file 1: Figure S3). The resulting power and false positive rates were worse than EIGENSTRAT.

### Performance study using empirical mouse genomes and simulated phenotypic data

**Fig. 7:**
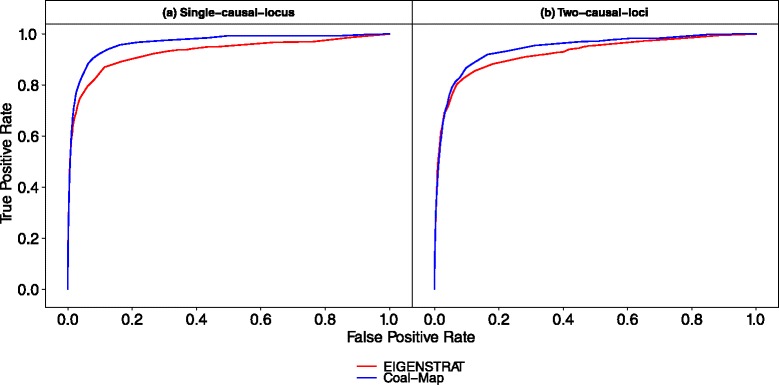
In the performance study utilizing genomic data from mouse chromosome 7, Coal-Map has similar or typically better power and type I error control compared to EIGENSTRAT. Figure layout and description are otherwise identical to Fig. 4. For the single-causal-locus model condition, Coal-Map and EIGENSTRAT have AUROC of 0.965 and 0.929, respectively; for the two-causal-loci model condition, Coal-Map and EIGENSTRAT have AUROC of 0.942 and 0.923, respectively

**Fig. 8:**
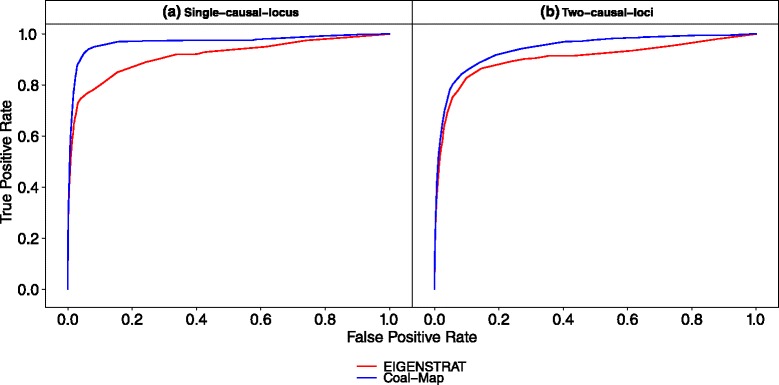
In the performance study utilizing genomic data from mouse chromosome 17, Coal-Map has similar or typically better power and type I error control compared to EIGENSTRAT. Figure layout and description are otherwise identical to Fig. 7. For the single-causal-locus model condition, Coal-Map and EIGENSTRAT have AUROC of 0.968 and 0.914, respectively; for the two-causal-loci model condition, Coal-Map and EIGENSTRAT have AUROC of 0.943 and 0.904, respectively

**Fig. 9:**
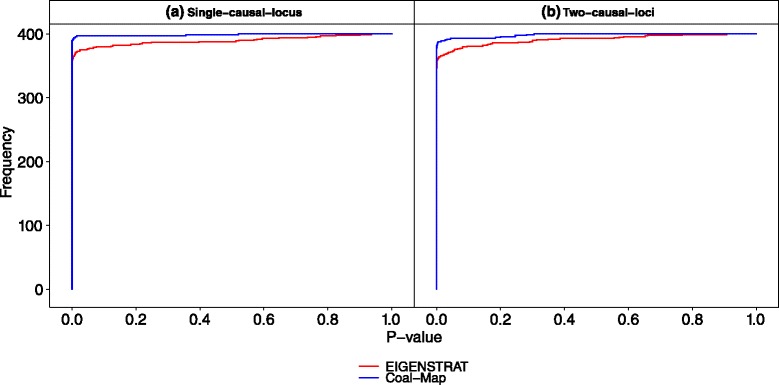
The cumulative histogram of *p*-values reported by Coal-Map and EIGENSTRAT at causal SNPs is shown for the performance study utilizing genomic data from mouse chromosome 7

**Fig. 10:**
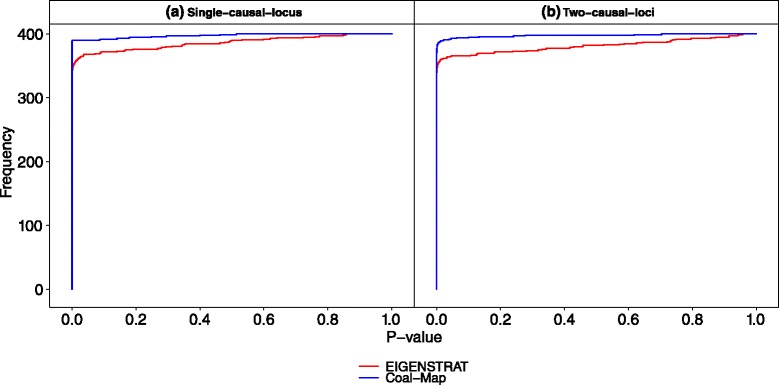
The cumulative histogram of *p*-values reported by Coal-Map and EIGENSTRAT at causal SNPs is shown for the performance study utilizing genomic data from mouse chromosome 17. Figure layout and description are otherwise identical to Fig. 9

Results from the performance study using empirical mouse genomes were consistent with the simulation study. Coal-Map’s AUROC was significantly better than EIGENSTRAT on chromosomes 7 and 17 (Table 2). (Delong et al. test [33], *α*=0.05). At an FPR of 0.05, Coal-Map’s TPR improvement over EIGENSTRAT was 0.076 and 0.166 for single-causal-locus traits, respectively, and 0.030 and 0.057 for two-causal-loci traits, respectively (Figs. 7 and 8). At causal SNPs in chromosomes 7 and 17, Coal-Map reported smaller *p*-values overall compared to EIGENSTRAT (Figs. 9 and 10, respectively). Compared to the performance study using synthetic genomes, the cumulative histograms were shifted down for both methods (i.e., fewer causal SNPs were inferred at each cutoff). We note that chromosomes 7 and 17 exhibited the greatest amount of introgression in our study (Additional file 1: Table S2). In contrast, chromosome 15 had the fewest number of introgressed sites in our study, with total length that was 68 % and 23 % smaller than those in chromosome 7 and 17, respectively; on this chromosome, the AUROC improvement of Coal-Map over EIGENSTRAT was weakly significant when mapping single-causal-locus traits and not significant for two-causal-loci traits (Table 2; Additional file 1: Figures S6 and S7).

**Table 2:** The performance of Coal-Map and EIGENSTRAT based on area under receiver operating characteristic curve (AUROC) is compared using empirical mouse chromosomes and simulated traits

Chromosome	Single-causal-locus			Two-causal-loci		
			Corrected			Corrected
	Coal-Map	EIGENSTRAT	q value	Coal-Map	EIGENSTRAT	q value
7	0.964	0.928	<10^−5^	0.942	0.923	0.003
15	0.940	0.922	0.014	0.917	0.919	0.587
17	0.968	0.914	<10^−5^	0.942	0.904	1.6∗10^−5^

On the two mouse chromosomes with the greatest number of introgressed sites in our study - chromosomes 7 and 17 - Coal-Map’s performance was significantly better than EIGENSTRAT for both single-causal-locus and two-causal-loci traits (Delong et al. test [33] with Benjamini-Hochberg correction [65]; setwise *α*=0.05; *n*=20 for each test). We observed a reduced performance improvement on chromosome 15, which had relatively fewer introgressed sites: the improvement was weakly significant for single-causal-locus traits and not significant for two-causal-loci traits (using the same test)

## Discussion

Our performance study utilized empirical and simulated data reflecting a wide range of evolutionary scenarios. The simulation conditions were based upon empirical studies of adaptive interspecific introgression. We consistently observed that Coal-Map had comparable or improved performance compared to EIGENSTRAT, a leading association mapping method in terms of its popularity, power, and type I error control.

One key factor that impacts Coal-Map’s performance is the genetic architecture of the trait under study. Coal-Map’s relative performance improvement is greatest on datasets with one or a few loci that contribute causal SNPs – which we refer to as causal loci – and is less on datasets with many causal loci. We hypothesize that the amount of local genealogical incongruence between causal loci is the main determining factor, not necessarily the number of causal loci. As incongruence becomes greater, the sample covariance contributed by any individual causal locus’s local sample structure will be diminished, and global sample structure (as measured across all sites in a dataset) will predominate. We note that, in prior genomic studies of adaptive introgression [3, 5], introgressed loci were observed to have similar sample structure (i.e., similar distributions of local genealogies). Introgression of alleles that were causal for an adaptive trait and genetic hitchhiking of neighboring loci were hypothesized to enhance genealogical congruence between loci with common introgressive origin. An empirical example is shown in Fig. 3. We therefore anticipate that, relative to other model conditions, the single-causal-locus and two-causal-loci model conditions in our study may be most relevant to the empirical study of adaptive introgression.

In our simulation study, we found that Coal-Map’s performance advantage was retained across a wide range of gene flow – even on model conditions which had virtually no gene flow. In the latter case, we attribute Coal-Map’s improvement to modeling the variation in local sample structure contributed by incomplete lineage sorting. The breakpoint inference stage of Coal-Map’s methodological pipeline accounts for local genealogical variation due to gene flow as well as incomplete lineage sorting and other evolutionary processes. We note that the presence of incomplete lineage sorting in our performance study is unique compared to past performance studies of association mapping methods on admixed populations (e.g., the study of Shriner et al. [34], which assumed that the parental populations contributing to an admixed population were completely unrelated). Compared to the synthetic genomes, the empirical genomes contained local genealogical variation due to an even wider array of evolutionary processes, including recombination. Crucially, the breakpoint inference stage of Coal-Map’s pipeline made use of PhyloNet-HMM, a probabilistic inference method that uses the coalescent model, phylogenetic networks, and hidden Markov models to account for all of these evolutionary processes acting in combination. Consistent with the simulation study, Coal-Map’s performance was comparable or better than EIGENSTRAT.

The choice of the number of covariates used to represent sample structure in Coal-Map was based upon a previous algorithmic design study examining the use of fixed effects models for association mapping [22]. To further explore the ramifications of this choice, we conducted an algorithmic design experiment to explore the impact of the number of covariates used in Coal-Map’s model upon its performance. We found that Coal-Map’s performance was robust to this design choice.

Coal-Map’s performance advantage over EIGENSTRAT was retained across different levels of environmental contribution to traits. A larger performance improvement was seen on model conditions with only a genotypic contribution to traits, which we ascribe to the lack of sample structure inherent in the additive environmental noise.

We observed that a model that only accounts for local sample structure resulted in reduced power and higher false positive rate compared to Coal-Map, which accounts for both local and global sample structure. Our finding is consistent with the findings of Shriner et al. [34]. The intuitive explanation is that local sample structure in the current partition (i.e., the partition enclosing the test SNP) should be modeled when the current partition contains causal SNPs, but otherwise not.

## Conclusions

Adaptive introgression involves the complex interplay of a variety of evolutionary processes including gene flow, directional selection, recombination, lineage sorting processes that may result in incomplete lineage sorting, and sequence mutation. The need to distinguish between these differing evolutionary forces in population genomic and comparative genomic studies was emphasized by two recent reviews [31, 35], and phylogenomic inference methods are actively being developed to study the interplay of these evolutionary processes [6, 11]. One of the genomic signatures of adaptive introgression is local genealogical variation featuring introgressed genomic tracts as long as dozens of megabases. These tracts contain introgressed loci of two types: adaptive loci and nearby linked neutral loci. Sample structure at these loci can differ greatly from global sample structure (i.e., sample structure measured across all sites). Traditional approaches to association mapping account only for the latter, assuming that sample structure is mostly invariant across the genome.

We therefore introduced Coal-Map, a new association mapping method which explicitly models both local sample structure, such as arises in a genomic region containing tracts of common introgressive origin, and global sample structure. Coal-Map is a methodological pipeline that incorporates recent theoretical innovations that bridge population-level evolution under the coalescent with traditional phylogenetic models of biomolecular sequence evolution [36, 37].

We validated the performance of Coal-Map using synthetic and empirical data. The data sets in our study featured local genealogical variation due to gene flow as well as incomplete lineage sorting, sequence mutation, and (in the case of the empirical mouse genomes) recombination. We compared the performance of Coal-Map to EIGENSTRAT, a leading association mapping method. We consistently observed the same outcome across all of the datasets in our study: Coal-Map’s performance in terms of power and false positive rate was comparable or better than EIGENSTRAT in all cases. Thus, Coal-Map can be generally used in place of EIGENSTRAT and related association mapping approaches that do not explicitly model local variation in evolutionary relatedness. We found that the conditions under which Coal-Map’s performance was strictly better than EIGENSTRAT were those that most closely resembled empirical cases of adaptive interspecific introgression, involving: (1) traits with causal SNPs drawn from one or a few genomic loci with common introgressive origin, and (2) a range of gene flow levels. Perhaps surprisingly, Coal-Map strictly outperformed EIGENSTRAT even on model conditions where gene flow was nearly absent but incomplete lineage sorting was still a factor. We hypothesize that modeling the fine variation in sample structure due to incomplete lineage sorting may be useful to association mapping approaches outside of the multi-species evolutionary context considered in this study. These alternative contexts include traditional genome-wide association studies of human populations or inbred mouse strains.

The pipeline-based design of Coal-Map is flexible. As noted above, richer coalescent-based modeling of the evolutionary origins of local genealogical variation may permit more accurate breakpoint inference in the first stage of Coal-Map.

We explored the use of forward selection for our model selection approach, which was used to determine if local sample structure containing the test SNP was incorporated into the model or not. However, we found that forward selection is conservatively biased towards models with fewer parameters. As an alternative, we utilized an approach involving two nested models – one incorporating local sample structure and the other not – which uses a likelihood ratio test of each model against a null model (positing that the test SNP has no effect), and then selects the model with the smallest test statistic. We note that, using this approach, the resulting association score is at least as significant as a likelihood ratio test using one of the two nested models by itself. Thus, the number of positives (and therefore the number of false positives) reported using the approach can be no smaller than the number reported using one of the two nested models, and the number of false negatives can be no larger. In practice, our results suggest that the approach yields a comparable false positive rate and a substantially improved true positive rate compared to forward selection and other model selection techniques such as information criteria [38–41].

Recently, Shriner et al. and Pasaniuc et al. proposed BMIX and MIXSCORE, two methodologies for genome-wide association studies of admixed populations [27, 28]. These methodologies combine admixture mapping – which makes use of ancestry effects of admixed loci – with traditional association mapping approaches – which makes use of genotypic effects without taking admixed ancestry into account – to yield improved mapping power and type I error control. Our study is orthogonal to their studies for the following reasons. First, both approaches assume that admixed populations arise due to admixture between two completely unrelated parental populations. In general, admixture mapping approaches make use of this assumption [42, 43]. This simplifies local ancestry inference since coalescence between lineages in the “pure” parental populations is not considered and therefore incomplete sorting of these lineages is not an issue, which could otherwise confound ancestry inference [31]. In reality, any two samples share a most recent common ancestor. Not accounting for coalescence between lineages can have first-order effects upon association mapping accuracy, which we demonstrate in our performance study. Notably, our study involves evolutionary divergence greater than those seen within a single species. In a traditional association mapping study of human populations or artificial lab strains of model organisms, the population split times are smaller than the divergence times in our study and thus incomplete lineage sorting should be more likely and have an even greater effect (assuming that effective population sizes are comparable or larger). Coal-Map relaxes the simplifying assumption of pure isolation using a coalescent-based approach. A recent review has highlighted the multispecies coalescent model as an ideal means to account for evolutionary relatedness at multiple scales in functional genomic studies [44]. Second, both approaches represent local sample structure differently from Coal-Map. Both BMIX and MIXSCORE assume that population strata are discrete due to pure isolation between parental populations. In contrast, we utilize continuous phylogenetic covariates to more flexibly represent evolutionary relatedness between samples. Third, BMIX utilizes stratified regression to perform association mapping (and recall that BMIX has been shown to yield more power and comparable false positive rate compared to MIXSCORE). In contrast, mixed model approaches to association mapping have been shown to out-perform stratified regression [17]. Coal-Map makes use of the latter approaches for this reason. Fourth, a primary contribution of the two studies is the insight that combining admixture mapping with association mapping out-performs association mapping by itself. The same insight can be applied to Coal-Map, yielding a combined approach that tests for introgressed ancestry effects (without regard to specific allele effects) in combination with genotypic effects. We hypothesize that a combined approach will yield further performance improvements compared to Coal-Map, similar to BMIX and stratified regression. Fifth, the problem domains differ greatly due to the disparate evolutionary divergences involved in the studies. Compared to intraspecific admixture, adaptive interspecific introgression involves a different set of evolutionary processes with first-order effects. While the study of adaptive traits of interspecific introgressive origin and their genomic architecture is of independent interest, the two settings may be complementary for studying certain biological questions. For example, some of the introgressed loci that contribute to warfarin resistance in mice have orthologs in human populations with related cardiovascular roles. Compared to human populations, natural mouse populations have undergone much stronger recent positive selection due to the introduction of rodenticide, which can be advantageous for association mapping purposes (e.g., stronger hitchhiking effects resulting in megabases-long introgressed tract lengths).

We conclude with discussion of future research directions. To our knowledge, our study is the first to explore association mapping performance using many hundreds of genomes with adaptive interspecific introgressive origins. Compared to human genome sequence data in public databases, a relative paucity of genomic sequence data is available to explore this question. Genomic sequence data is available for less than a thousand samples from natural populations of house mice [45–47], and even less phenotypic data is available for introgressed mouse populations. The situation is similar in studies of other organisms. For example, in the studies of Counterman et al. [48] and Baxter et al. [49], approximately a hundred samples were used to examine adaptive introgression between butterfly species and its role in wing pattern mimicry. We anticipate that recent biotechnological advances will remove this limitation in the near term, especially as researchers continue to investigate adaptive interspecific introgression in eukaryotes. We also plan on exploring several aspects of Coal-Map’s algorithmic design in future work. First, other model selection strategies can be used in a Coal-Map analysis (as noted above). In particular, cross-validation has been shown to perform well for model selection problems in phylogenomics [50]. This approach requires a relatively greater amount of data compared to the strategies explored in this study. Second, following MultiBLUP and Adaptive MultiBLUP’s approaches to a different computational problem [44], search techniques can be used to find multiple causal loci. We anticipate two main challenges with the use of more complex models as part of a search-based approach: (1) model selection strategies will be necessary to distinguish between models that differ in the number of parameters needed to capture genetic effects from multiple causal loci, and (2) efficient techniques will be needed to learn a potentially large number of model parameters. Third, global and local sample structure could be modeled using random effects instead of fixed effects. Software libraries for generalized linear models with multiple random effects are available for this purpose [24, 44]. Fourth, a combined approach that simultaneously performs introgression breakpoint inference, local ancestry mapping, and association mapping that accounts for local sample structure may offer additional performance improvements beyond those observed in our study. Finally, Zuk et al. [51] note that one important research direction is relaxing the assumption of additive genotypic contribution to complex traits. We share their opinion that the future of association mapping will involve improved modeling of epistasis and complex traits with polygenic architectures. A promising future direction is to model multiple effects at multiple scales of systems biology [52], such as examining dependence between genotypic characters, interactomic graphs, and phenotypic characters.

## Methods

### Coal-Map

The input to Coal-Map consists of: (1) an *n* by *k* multiple sequence alignment ***X*** containing genotypic data for *n* aligned sequences and *k* sites, (2) local partition breakpoints ***b*** in ascending order (including trivial breakpoints corresponding to sites 1 and *k*), and (3) a phenotypic vector ***y*** with *n* observations. The *i*th row in alignment ***X*** and the *i*th entry in the phenotypic vector ***y*** correspond to the genomic sequence and phenotypic value of taxon *s*_*i*_ for 1≤*i*≤*n*. A local partition ***X***_***ℓ***_ is the alignment consisting of all sites in alignment ***X*** contained in the closed interval with endpoints equal to the breakpoints *b*_*ℓ*_ and *b*_*ℓ*+1_. Each local partition ***X***_***ℓ***_ represents a contiguous genomic region where introgressed tracts in the region had a common introgressive origin (i.e., originated from lineages that evolved within the same network edges in the species phylogeny $\mathcal {N}$), as inferred by either Phylonet-HMM [11] in the general case or a simpler parsimony-based alternative when certain assumptions were satisfied (see below). The output of Coal-Map consists of a score *p*_*j*_ that measures the statistical association between the genotypic character ***x***_***j***_ (i.e., the *j*th column in *X*) and phenotypic vector ***y*** for 1≤*j*≤*k*.

The association score *p*_*j*_ for a SNP ***x***_***j***_ is calculated under the following linear mixed model (following the notation of [23]):

$$\begin{array}{*{20}l} \boldsymbol{y} &= \boldsymbol{W_{j}}\boldsymbol{\alpha} + \boldsymbol{x_{j}} \beta + \boldsymbol{\epsilon} \\ \boldsymbol{\epsilon} &\sim {MVN}_{n} (0, \tau^{-1} \boldsymbol{I_{n}}) \end{array} $$

The test SNP ***x***_***j***_ has effect size *β*. The fixed effects ***W***_***j***_ includes covariates that account for global sample structure (i.e., sample structure measured across all sites in alignment ***X***). Additional covariates that account for local sample structure (i.e., sample structure inferred within the local partition ***X***_***ℓ***_ containing the test SNP ***x***_***j***_) may be added using a model selection approach (described below). The covariates have coefficients ***α***. The error term ***ε*** follows an *n*-dimensional multivariate normal distribution with mean 0 and variance *τ*^−1^***I***_***n***_. We obtained MLE estimates $\hat {\boldsymbol {\alpha }}$, $\hat {\beta }$, and $\hat {\tau }$ using the optimization procedure described in Supplementary Note 3.1.1 from [23]. The association test score *p*_*j*_ is computed using a likelihood-ratio test of the fitted model against a null model of no SNP effect.

We chose to model sample structure using fixed effects instead of random effects due to the popularity of fixed-effect association mapping approaches and their accuracy [19, 20, 22, 34, 53]. Global sample structure is represented using covariates ***W***_***global***_, obtained as follows. Following the approach of [22], a principal components analysis is performed on the full alignment *X* excluding the local partition ***X***_***ℓ***_ and the top five principal components are used as the covariates ***w***_***1***_,***w***_***2***_,…,***w***_***5***_. Local sample structure in a local partition ***X***_***ℓ***_ containing the test SNP *x*_*j*_ is represented similarly, resulting in five additional covariates ***w***_***5***_,***w***_***6***_,…,***w***_***10***_. We chose to use at most ten covariates to represent sample structure based on the empirical findings of [22]. We also evaluated the sensitivity of Coal-Map to the number of covariates using five and twenty covariates.

The basic idea behind our use of fixed covariates to capture sample relatedness is as follows. Causal loci outside of the local partition ***X***_***ℓ***_ (containing the test SNP) are modeled as a polygenic effect based on covariates computed from global sample structure since it aggregates over all partitions. Covariates computed from local sample structure contribute to the linear mixed model when the local partition ***X***_***ℓ***_ contributes to the phenotypic vector ***y*** (i.e., contains causal SNPs), but otherwise not. Coal-Map selects between the two resulting models: one using the covariates $\boldsymbol {W_{j}^{\text {global}}} = (\boldsymbol {w_{1}}, \boldsymbol {w_{2}}, \ldots, \boldsymbol {w_{5}})$ and the other using the covariates $\boldsymbol {W_{j}^{\text {glocal}}} = (\boldsymbol {w_{1}}, \boldsymbol {w_{2}}, \ldots, \boldsymbol {w_{10}})$. In this study we explore the use of two approaches for this task. Since the two models are nested, we use a forward selection approach (significance threshold of *P*<0.05). We also evaluated an alternative approach which performs the likelihood-ratio test of each alternative model against a null model of no SNP effects and then chooses the model with smaller *P* value. Since the forward selection approach can be conservative, we focus on the latter approach.

### Simulation study

Our performance evaluation of Coal-Map utilized simulated genotypic sequences and traits where local genealogical variation was due to gene flow and incomplete lineage sorting, two evolutionary processes with first-order effects in prior studies of introgressed eukaryotic genomes [1–3, 5, 31, 54]. The genotypic sequence data was simulated under the basic coalescent model [32] with instantaneous admixture [10] and a bi-allelic sequence mutation model. Simulations were run using either msms [55] to perform forward-time simulation that explicitly modeled positive selection or ms [56] to perform backward-time simulation with intra-locus linkage to emulate the genomic patterns of positive selection. Based on the the analytical and empirical findings of Neuhauser and Krone [57], both simulation approaches are expected to generate similar patterns of local genealogical variation. Furthermore, our performance study obtained consistent results for both approaches. The ms-based simulation utilized an infinite sites model of sequence mutation; the msms-based simulation utilized a sequence mutation model that allowed recurrent mutations between two alleles. Our forward-time coalescent simulation used a selection coefficient *s*=0.56 which was based upon previously reported estimates from natural mouse populations that were involved in adaptive introgression [4]. These populations are represented by empirical samples used in our study (see below). The model phylogeny used for simulation is shown in Fig. 2. Two present-day populations A and B diverged from a most recent common ancestral population at time *t*_1_. At time *t*_2_, the ancestral populations of A and B hybridized to form the ancestral population of H. We based the divergence time *t*_1_=3.0 (in coalescent units) on prior population genetic estimates in two *Mus* species (see Table 1 in [11]), corresponding to a divergence time of 1.5 Mya bp, generation time of 2 generations per year, and an effective population size of *N*_*e*_=2.5∗10^5^. We explored hybridization frequency values consisting of *γ*∈{0.5,0.25,0.1,0.01} in the backward-time coalescent simulations, *γ*=0.5 in the forward-time coalescent simulations, mutation rate *μ*=1.0 in the forward-time coalescent simulations, and time *t*_2_=2.0. Each dataset consisted of 10 loci sampled from the above model with free recombination between loci and complete linkage of sites within each locus. We used a sequence length of 250 bp/locus, resulting in 2.5 kb per simulated multiple sequence alignment. Based on the simplifying assumptions of our simulation study (infinite sites model, free recombination between loci, and complete linkage within each locus), the local partition breakpoints required as input to Coal-Map were inferred using the Four-Gamete Test [58, 59].

For each multiple sequence alignment, a quantitative trait was simulated under an extended version of the additive model used by [60] and [61], which incorporated polygenic contributions from multiple loci as follows:

$$ y_{i} = \pi \sum\limits_{j \in \Delta}\frac{Q_{i,j}}{|\Delta|} + (1-\pi) N(0,0.01), $$

where *y*_*i*_ is the trait value for the *i*th individual, *π* is the proportion of trait variation contributed by genotypic effects, *Q*_*i*,*j*_ is 1 if the *i*th individual carries the derived allele at site *j* or 0 otherwise, the environmental contribution is a random effect with normal distribution *N*(0,0.01), and *Δ* is the set of causal SNPs. Twenty causal SNPs were selected uniformly at random from either one, two, or all loci, where loci were chosen uniformly at random in the case of a single or two loci contributing causal SNPs. We refer to the resulting model conditions as single-causal-locus, two-causal-loci, and all-causal-loci model conditions, respectively. Additionally, causal SNPs were selected to have minor allele frequency ranging between 0.1 and 0.3 (following the cutoffs used in [17]). In the forward-time coalescent simulations, loci containing causal markers were under positive selection and all other loci evolved in a neutral manner. For each model condition, simulation was repeated twenty times, resulting in twenty replicate datasets per model condition. Each replicate dataset was analyzed using Coal-Map and EIGENSTRAT. EIGENSTRAT was run with default settings, where the covariates include the top ten principal components computed from the alignment ***X***.

### Performance study using empirical mouse genomes

To better understand the performance of Coal-Map in the context of adaptive interspecific introgression, our performance study utilized genomic sequence data from a past study of genetic variation in natural *Mus musculus* and *Mus spretus* populations [5]. Detailed sample information for the 744 haploid mouse genomes is shown in Additional file 1: Table S1. The sequencing procedures were described in [5], which we briefly review here. Genomic sequence data came from two sources: (1) wild and wild-derived samples from prior studies [4, 5, 47, 62, 63] that were genotyped using the Mouse Diversity Array (following the procedure of [63]) and phased into haploid sequences, and (2) wild-derived samples with published whole-genome sequences [45]. The sequences were combined and then filtered to focus on 414,376 SNPs that were genotyped in all samples. We analyzed the genomic sequences using PhyloNet-HMM to infer genomic tracts with interspecific introgressive origin, following the procedure of [5]. Figure 3 shows the genomic tracts in the vicinity of the *Vkorc1* gene that PhyloNet-HMM inferred as introgressed in origin. Crucially, PhyloNet-HMM performs probabilistic inference to ascribe local genealogical variation to one of several evolutionary processes: interspecific introgression, incomplete lineage sorting, recombination, back mutation, and any combination thereof. Thus, local sample structure will often vary within introgressed tracts that are shared across a common subset of samples (cf. Fig. 10 in [11]).

Two main factors affecting association mapping power are effect size and minor allele frequency of causal SNPs [64] – both of which are unknown a priori. We therefore conducted a performance study using synthetic quantitative traits where these factors were specified as model parameters. We used the above trait model with genetic contribution from introgressed genomic tracts associated with the introduction of warfarin pesticide in Europe [4, 5]. Local partition breakpoints were based upon the union of the PhyloNet-HMM-inferred introgressed tracts and causal SNPs were selected from local partitions where only the genomes of rodenticide-resistant mice had introgressed tracts and contained at least 100 sites. We focused on mouse chromosomes with at least two such partitions, which consisted of chromosomes 7, 15, and 17. For each chromosome, single-causal-locus trait simulation was repeated to yield twenty replicate datasets, and similarly for two-causal-loci traits. The empirical genomic sequence data, synthetic trait data, and local partition breakpoints were provided to Coal-Map as inputs.

## Availability of supporting data

Open-source software and open data are available at gitlab.msu.edu/liulab/coal-map.

## Additional file

Additional file 1
**Appendix.** Appendix, including text, tables, and figures for performance study using empirical genomic sequence data from mouse chromosome 15, information about mouse samples used in the study, and supplementary experiments related to the algorithmic design of Coal-Map. (PDF 289 kb)
