# Erratum to: ‘Mapping the genomic architecture of adaptive traits with interspecific introgressive origin: a coalescent-based approach’

**DOI:** 10.1186/s12864-016-2497-5

**Published:** 2016-04-18

**Authors:** Hussein A. Hejase, Kevin J. Liu

**Affiliations:** Department of Computer Science and Engineering, Michigan State University, 428 S. Shaw Lane, 48824 East Lansing, MI USA

Unfortunately, the original version of this article [1] contained an error. The figures was included incorrectly. The correct figures and their captions can be found below:
