# Supplementary material for: Mapping the genomic architecture of adaptive traits with interspecific introgressive origin: a coalescent-based approach
Source: BMC Genomics. 2016 Jan 11;17(Suppl 1):8. doi: 10.1186/s12864-015-2298-2 (PMC4895787; doi:10.1186/s12864-015-2298-2)
Supplement: Additional file 1 — Appendix. Appendix, including text, tables, and figures for performance study using empirical genomic sequence data from mouse chromosome 15, information about mouse samples used in the study, and supplementary experiments related to the algorithmic design of Coal-Map. (PDF 289 kb) [file 12864_2015_2298_MOESM1_ESM.pdf]

# Appendix

Hussein A. Hejase & Kevin J. Liu

## 1 Varying the number of principal components for modeling sample structure

We examined the sensitivity of Coal-Map to the number of covariates used to model global and local sample structures. First, we represented the local sample structure using the top three covariates obtained after applying principal components analysis on the local partition $X_l$ containing the test locus $x_j$. Global sample structure was represented using the top two covariates after performing principal components analysis on the full alignment $X$ excluding the local partition $X_\ell$. This resulted in two models: one using the covariates $W_j^{\text{global}} = (w_1, w_2)$ and the other using the covariates $W_j^{\text{glocal}} = (w_1, w_2 \ldots w_5)$, respectively. We selected one of the aforementioned two models for each test locus $x_j$ using the heuristic approach described in the Methods section. In Figure S1, the performance of Coal-Map, using five covariates to represent sample structure (two for global and three for local), and EIGENSTRAT is shown using receiver operating characteristic (ROC) curves. Using Delong *et al.* test [1] with Benjamini-

Hochberg correction [2], Coal-Map's performance improvement was significantly greater than EIGENSTRAT in terms of area-under-ROC-curve (AUROC) for the single-causal-marker (q value $< 10^{-5}$) and two-causal-marker (q value $< 10^{-5}$) model conditions but not for the all-causal-marker model condition (q value $= 0.26$). At a false positive rate (FPR) of 0.05, Coal-Map's true positive rate (TPR) improved upon EIGENSTRAT's by 0.230 and 0.079 on the single-causal-marker and two-causal-marker model conditions, respectively; on the all-causal-marker model condition, the TPR difference between the two methods was less than 0.001.

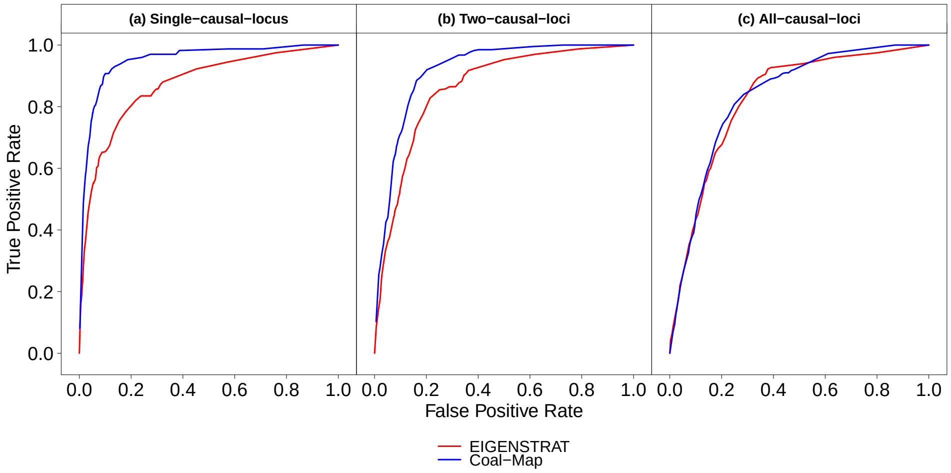


Figure S1: **Compared to EIGENSTRAT, Coal-Map using two global covariates and three local covariates has comparable or better power and false positive rate on model conditions with hybridization frequency $\gamma = 0.5$.** True positive rate and false positive rate are shown for both methods using receiver operating characteristic (ROC) curves. (a) Results are shown for the model condition where causal loci are drawn from a single marker. Coal-Map and EIGENSTRAT have area-under-ROC-curve (AUROC) of 0.949 and 0.871, respectively. (b) Results are shown for the two-causal-marker model condition. Coal-Map has an AUROC of 0.916 and EIGENSTRAT has an AUROC of 0.861. (c) Results are shown for the all-causal-marker model condition, where Coal-Map and EIGENSTRAT have AUROC of 0.833 and 0.827, respectively.

We further examined the performance of Coal-Map using ten covariates to represent

the global sample structure and ten covariates to represent the local sample structure. Figure S2 shows the performance of Coal-Map, using twenty covariates to represent sample structure (ten for global and ten for local), and EIGENSTRAT using ROC curves. Using Delong *et al.* test [1] with Benjamini-Hochberg correction [2], Coal-Map's performance improvement was significantly greater than EIGENSTRAT in terms of AUROC for the single-causal-marker (q value $< 10^{-5}$) and two-causal-marker (q value $< 10^{-5}$) model conditions but not for the all-causal-marker model condition (q value $= 0.26$). At a FPR of 0.05, Coal-Map's TPR improved upon EIGENSTRAT's by 0.115 and 0.082 on the single-causal-marker and two-causal-marker model conditions, respectively; on the all-causal-marker model condition, the TPR difference between the two methods was less than 0.001.

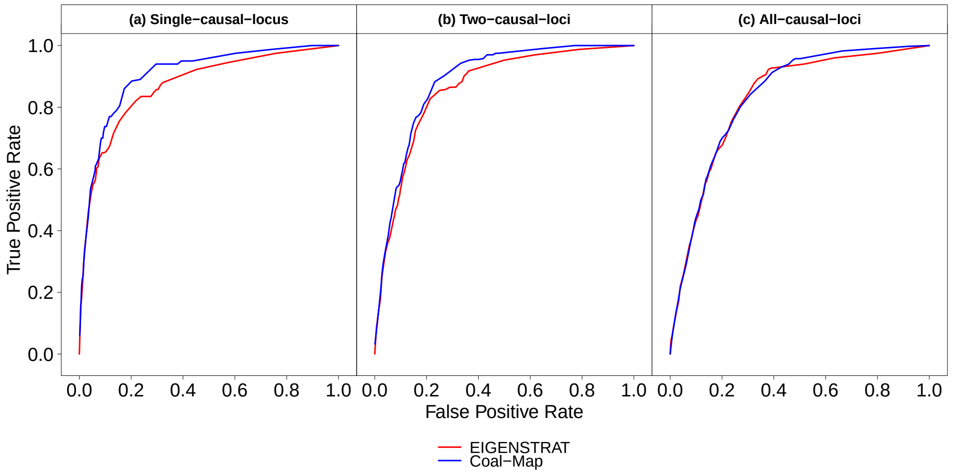


Figure S2: **Compared to EIGENSTRAT, Coal-Map using ten global covariates and ten local covariates has comparable or better power and false positive rate on model conditions with hybridization frequency $\gamma = 0.5$.** Figure layout and description are otherwise identical to Figure S1. (a) Results are shown for the model condition where causal loci are drawn from a single marker. Coal-Map and EIGENSTRAT have AUROC of 0.904 and 0.871, respectively. (b) Results are shown for the two-causal-marker model condition. Coal-Map has an AUROC of 0.884 and EIGENSTRAT has an AUROC of 0.861. (c) Results are shown for the all-causal-marker model condition, where Coal-Map and EIGENSTRAT have AUROC of 0.833 and 0.826, respectively.

## 2 A partitioned approach that only accounts for local sample structure

We compared the performance of EIGENSTRAT to a partitioned approach that only accounts for local sample structure using coalescent simulations (Figure S3). For a single-causal-marker model condition, the partitioned approach had similar performance to EIGENSTRAT where the AUROC improvement of the partitioned approach over EIGENSTRAT was not significant (p-value = 0.46). For the two-causal marker

model condition, the partitioned approach was outperformed by EIGENSTRAT. At a FPR of 0.05, the partitioned approach TPR improvement over EIGENSTRAT was less than 0.03 and 0.04 for single-causal-marker and two-causal-marker model conditions, respectively. This result highlights the importance of accounting for both local and global sample structures in association mapping studies.

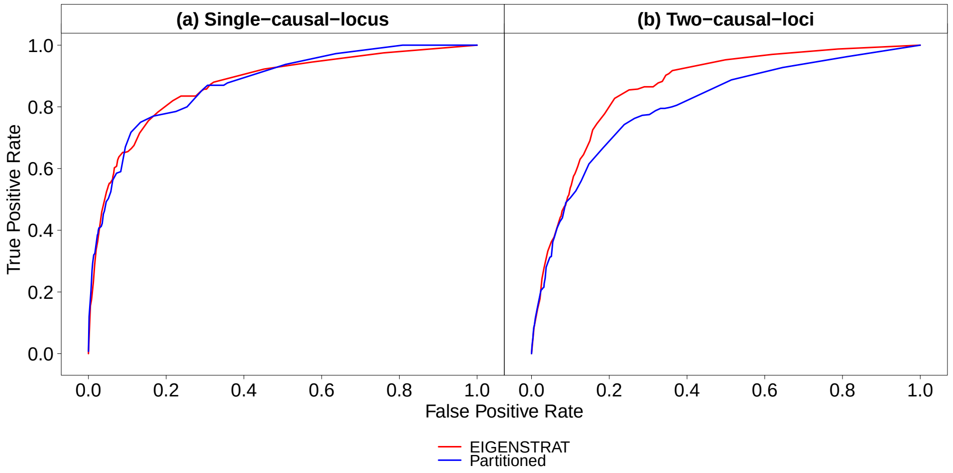


Figure S3: **Compared to a partitioned approach that only accounts for local sample structure, EIGENSTRAT has equal or better power and similar type I error control on the simulated datasets ($\gamma = 0.5$).** Figure layout and description are otherwise similar to Figure S1. (a) Results are shown for the model condition where causal loci are drawn from a single marker. Coal-Map and EIGENSTRAT have an AUROC of 0.873 and 0.870, respectively. (b) Results are shown for the two-causal-marker model condition, where Coal-Map and EIGENSTRAT have AUROC of 0.804 and 0.860, respectively.

# 3 Trait model with no environmental effect

Using a trait model that only accounts for the genotypic component with no environmental effect, Coal-Map performs better than EIGENSTRAT using single-causal-marker and two-causal-marker model conditions (Figures S4). Coal-Map's AUROC

improvement over EIGENSTRAT was significant using single-causal-marker (q value $< 10^{-5}$) and two-causal-marker (q value $< 10^{-5}$) model conditions. At a FPR of 0.05, Coal-Map's TPR improvement over EIGENSTRAT was 0.256 and 0.139 for single-causal-marker and two-causal-marker model conditions, respectively. This is greater by 0.145 and 0.055 for single-causal-marker and two-causal-marker compared to results obtained by Coal-Map with a trait model that accounts for both genotypic and environmental components.

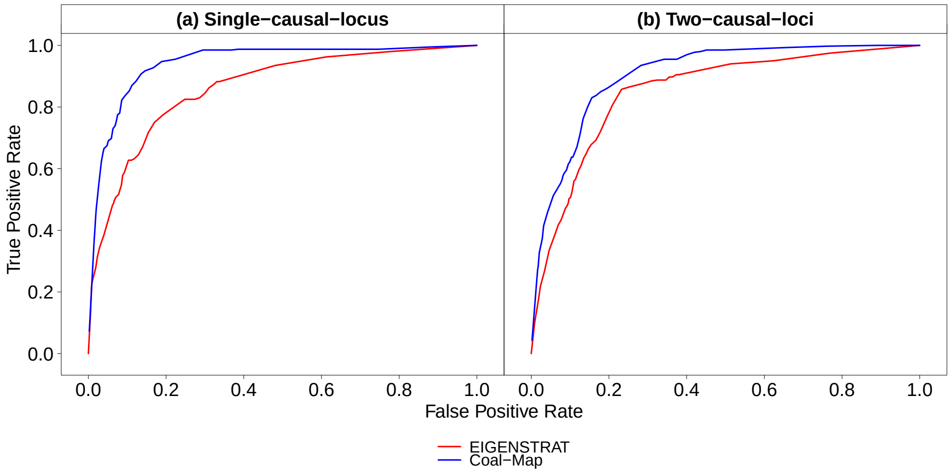


Figure S4: **Compared to EIGENSTRAT, Coal-Map has typically better power and similar type I error control on the simulated datasets with no environmental effect ($\gamma = 0.5$).** Figure layout and description are otherwise similar to Figure S1. (a) Results are shown for the model condition where causal loci are drawn from a single marker. Coal-Map and EIGENSTRAT have an AUROC of 0.942 and 0.860, respectively. (b) Results are shown for the two-causal-marker model condition, where Coal-Map and EIGENSTRAT have AUROC of 0.903 and 0.849, respectively.

# 4 Model selection

We performed model selection using a forward selection approach. This model selection approach is conservative and biased towards selecting the model with fewer parameters.

In our simulation study, the forward selection approach was biased towards selecting $W_j^{\text{global}}$, which is the model that only represents the global sample structure. Figure S5 shows the performance of Coal-Map and EIGENSTRAT using ROC curves. Using Delong *et al.* test [1] with Benjamini-Hochberg correction [2], Coal-Map's performance improvement was significantly greater than EIGENSTRAT in terms of AUROC for the single-causal-marker (q value $< 10^{-5}$) and two-causal-marker (q value $= 0.0002$) model conditions but not for the all-causal-marker model condition (q value $= 0.83$).

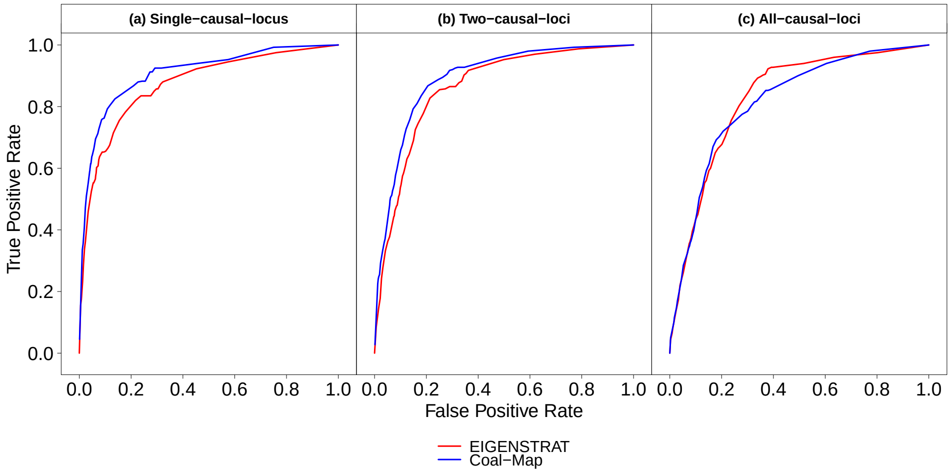


Figure S5: **Compared to EIGENSTRAT, Coal-Map using the forward selection approach has comparable or slightly better power and false positive rate on model conditions with hybridization frequency $\gamma = 0.5$.** Figure layout and description are otherwise identical to Figure S1. (a) Results are shown for the model condition where causal loci are drawn from a single marker. Coal-Map and EIGENSTRAT have AUROC of 0.907 and 0.871, respectively. (b) Results are shown for the two-causal-marker model condition. Coal-Map has an AUROC of 0.890 and EIGENSTRAT has an AUROC of 0.861. (c) Results are shown for the all-causal-marker model condition, where Coal-Map and EIGENSTRAT have AUROC of 0.814 and 0.827, respectively.

# 5 Performance study using empirical data from mouse chromosome 15

Comparing the performance of Coal-Map and EIGENSTRAT across chromosome 15 using empirical mice genomes, Coal-Map performs slightly better than EIGENSTRAT using single-causal-marker model condition where the AUROC improvement is weakly significant (q value = 0.014). For two-causal-marker model condition, Coal-Map has a similar performance to EIGENSTRAT where the AUROC improvement of Coal-Map over EIGENSTRAT is not significant (q value = 0.585). At a FPR of 0.05, Coal-Map's TPR improvement over EIGENSTRAT was less than 0.03 and 0.05 for single-causal-marker and two-causal-marker model conditions, respectively (Figures S6). At causal loci in chromosome 15, Coal-Map reported smaller p-values overall compared to EIGENSTRAT (Figure S7).

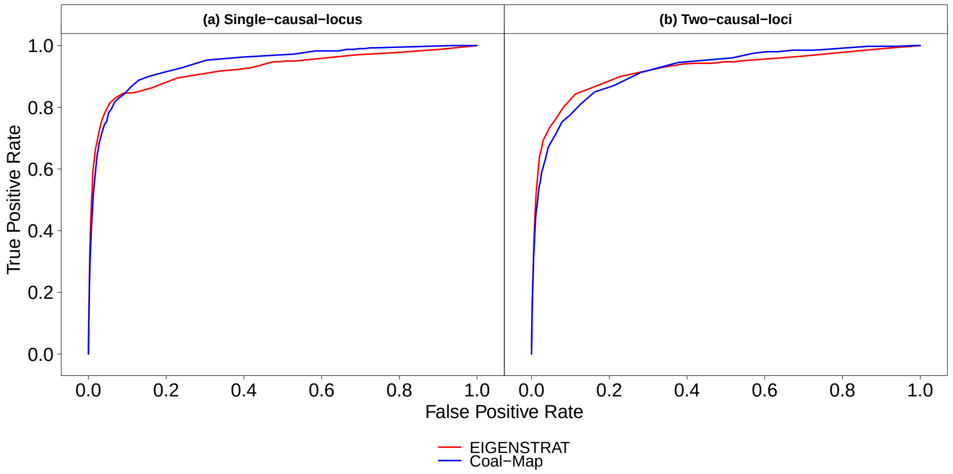


Figure S6: **In the performance study utilizing genomic data from mouse chromosome 15, Coal-Map has comparable power and similar type I error control compared to EIGENSTRAT.** Figure layout and description are otherwise similar to Figure S1. For the single-causal-marker model condition, Coal-Map and EIGENSTRAT have an AUROC of 0.940 and 0.922, respectively; for the two-causal-marker model condition, Coal-Map and EIGENSTRAT have AUROC of 0.917 and 0.919, respectively.

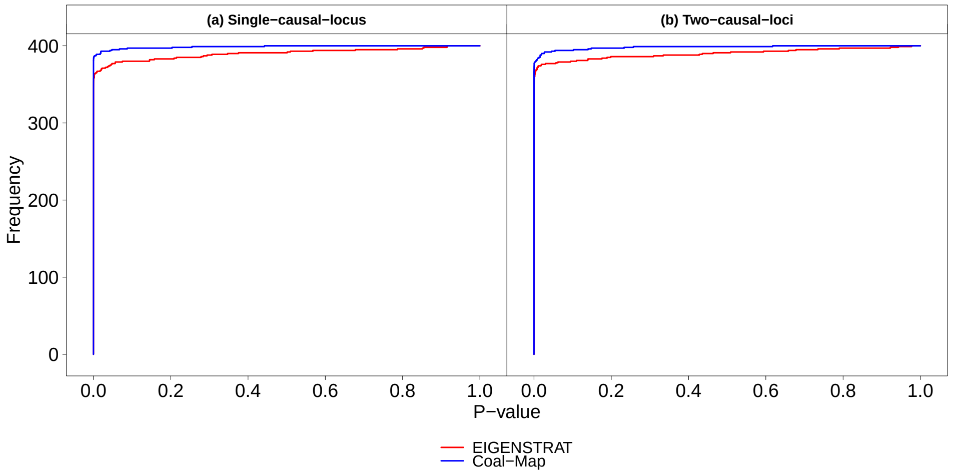


Figure S7: **The cumulative histogram of p-values reported by Coal-Map and EIGENSTRAT at causal loci is shown for the performance study utilizing genomic data from mouse chromosome 15.** Results are shown for the (a) single-causal-marker and (b) two-causal-marker model conditions, respectively. Cumulative frequency is reported over all replicates from a model condition.

# 6 Empirical sample information

Table S1 reports the detailed sample information along with their type and origin. Using these mice samples, PhyloNet-HMM inferred the introgressed tracts in chromosome 7, 15, and 17. Table S2 reports the identified introgressed tracts across the aforementioned chromosomes along with their start and end coordinates, and length of each introgressed tract.

| Sample name | Type (Origin) |
|---|---|
| B9 | Wild caught (Hamm, North Rhine-Westphalia, Germany) |
| B10 | Wild caught (Hamm, North Rhine-Westphalia, Germany) |
| B11 | Wild caught (Hamm, North Rhine-Westphalia, Germany) |
| BAG3 | Wild caught (Bukovce, Slovak Republic) |
| BAG56 | Wild caught (Pomykow, Lublin, Poland) |
| BAG68 | Wild caught (Wola Duza, Lublin, Poland) |
| BAG74 | Wild caught (Krasne, Podkarpackie, Poland) |
| BAG94 | Wild caught (Szepes, Debrecen, Hajdu-Bihar, Hungary) |
| BAG99 | Wild caught (Szomolyom, Hajdu-Bihar, Hungary) |
| BAG102 | Wild caught (Gabortelep, Bekes, Hungary) |
| C1 | Wild caught (Hamm, North Rhine-Westphalia, Germany) |
| C2 | Wild caught (Hamm, North Rhine-Westphalia, Germany) |
| C3 | Wild caught (Hamm, North Rhine-Westphalia, Germany) |
| KCT222 | Wild caught (Remderoda, Germany) |
| MWN1026 | Wild caught (San Girogio, Curone Valley, Piamonte, Italy) |
| MWN1030 | Wild caught (Menconico, Staffora Valley, Lombardia, Italy) |
| MWN1106 | Wild caught (Cassino, Lazio, Italy) |
| MWN1194 | Wild caught (Korinthos, Velo, Peleponissos, Greece) |
| MWN1198 | Wild caught (Laganas, Zakinthos Island, Greece) |
| MWN1214 | Wild caught (Milazzo, Olivarella, Sicily, taly) |
| MWN1279 | Wild caught (Arel, Mallorca island, Spain) |
| MWN1287 | Wild caught (Roca del Valles, Catalunya, Spain) |
| RDS10105 | Wild caught (Monchhof, Austria) |
| RDS12763 | Wild caught (Tubingen, Germany) |
| RDS13554 | Wild caught (Hubinger-Leitham, Austria) |
| SPRET/EiJ | Wild caught (Puerto Real, Cadiz Province, Spain) |
| Yu2097m | Wild caught (Urumqi, Xinjiang, China) |
| Yu2099f | Wild caught (Urumqi, Xinjiang, China) |
| Yu2115m | Wild caught (Yutian, Xinjiang, China) |

| | |
|---|---|
| Yu2120f | Wild caught (Hebukesaier, Xinjiang, China) |
| DCP | Wild-derived laboratory strain (Paphos, Cyprus) |
| STUS | Wild-derived laboratory strain (Studenec, Moravia, Czech Repuplic) |
| MOLG/DnJ | Wild-derived laboratory strain (Fukuoka, Kyushu, Japan) |
| BIK/g1 | Wild-derived laboratory strain (Kefar Galim, Israel) |
| BULS | Wild-derived laboratory strain (Buskovice, Bohemia, Czech Republic) |
| MDH | Wild-derived laboratory strain (Hov, Denmark) |
| PERA/EiJ | Wild-derived laboratory strain (Nana Village, Rimac Valley, Peru) |
| SOD1/EiJ | Wild-derived laboratory strain (Greece) |
| BUSNA | Wild-derived laboratory strain (Buskovice, Bohemia, Czech Republic) |
| PERC/EiJ | Wild-derived laboratory strain (Nana Village, Rimac Valley, Peru) |
| STRA | Wild-derived laboratory strain (Straas, Bavaria, Germany) |
| DEB | Wild-derived laboratory strain (Barcelona, Spain) |
| C57BL/6J | Wild-derived laboratory strain |
| STRB | Wild-derived laboratory strain (Straas, Bavaria, Germany) |
| SF/CAMEiJ | Wild-derived laboratory strain (California, US) |
| MSM/Ms | Wild-derived laboratory strain (Mishima city, Shizuoka Prefecture, Japan) |
| STLT | Wild-derived laboratory strain (Straas, Bavaria, Germany) |
| SKIVE/EiJ | Wild-derived laboratory strain (Skive, Denmark) |
| DCA | Wild-derived laboratory strain (Akrotiri, Cyprus) |
| CAST/EiJ | Wild-derived laboratory strain (Thonburi, Thailand) |
| RBF/DnJ | Wild-derived laboratory strain (Valle di Poschiavo, Switzerland) |
| MGA | Wild-derived laboratory strain (F Bonhomme, Alazani) |
| POHN | Wild-derived laboratory strain |
| MBS | Wild-derived laboratory strain (F Bonhomme, Sokolovo) |
| MPB | Wild-derived laboratory strain (F Bonhomme, Bialowieza) |
| MBK | Wild-derived laboratory strain (F Bonhomme, Kranevo) |
| CZECHI/EiJ | Wild-derived laboratory strain (Studenec, Moravia, Czech Republic) |
| MBT | Wild-derived laboratory strain |
| IS/CamRK | Wild-derived laboratory strain (Israeli port, Israel) |
| MH | Wild-derived laboratory strain (Hungary) |
| MDGI | Wild-derived laboratory strain (Givskud, Denmark) |
| WSB/EiJ | Wild-derived laboratory strain (Centerville, Maryland, US) |
| ZALENDE/EiJ | Wild-derived laboratory strain (Zalende, Switzerland) |
| MCZ | Wild-derived laboratory strain (Zitec, Czech Repuplic) |
| TIRANO/EiJ | Wild-derived laboratory strain (Tirano Upper Valtellina Valley, Lombardia, Italy) |
| BZO | Wild-derived laboratory strain (Oran, Algeria) |
| RBB/DnJ | Wild-derived laboratory strain (Bondo, Val Bregaglia, Graubunden) |
| STUP | Wild-derived laboratory strain (Studenec, Moravia, Czech Repuplic) |
| MOLD/RkJ | Wild-derived laboratory strain (Fukuoka, Kyushu, Japan) |
| STUF | Wild-derived laboratory strain (Studenec, Moravia, Czech Repuplic) |
| JF1/Ms | Wild-derived laboratory strain (JAX, Denmark Market) |

| | |
|---|---|
| DDO | Wild-derived laboratory strain (Odis, Denmark) |
| PANCEVO/EiJ | Wild-derived laboratory strain (Pancevo, Serbia) |
| DOT | Wild-derived laboratory strain (Tahiti, Oceania) |
| RBA/DnJ | Wild-derived laboratory strain (Mutten Albula Valley, Graubunden, Switzerland) |
| DJO | Wild-derived laboratory strain (Orcetto, Italy) |
| MOR/RkJ | Wild-derived laboratory strain (Ohio, US) |
| CTP | Wild-derived laboratory strain |
| WMP | Wild-derived laboratory strain (Monastir, Tunisia) |
| 22MO | Wild-derived laboratory strain (Monastir, Tunisia) |
| CIM1 | Wild-derived laboratory strain (Masinagudi, India) |
| MOLF/EiJ | Wild-derived laboratory strain (Fukuoka, Kyushu, Japan) |
| WLA | Wild-derived laboratory strain (Toulouse , France) |
| LEWES/EiJ | Wild-derived laboratory strain (Delaware, US) |
| DMZ | Wild-derived laboratory strain (Azemmour, Moroco) |
| PWK/PhJ | Wild-derived laboratory strain (Lhotka, Bohemia, Czech Repuplic) |
| PWD/PhJ | Wild-derived laboratory strain (Kunratice near Prague, Bohemia, Czech Repuplic) |
| CZECHII/EiJ | Wild-derived laboratory strain (Bratislava, Slovak Republic) |
| PWK hybrid | Wild-derived laboratory strain |
| CALB/RkJ | Wild-derived laboratory strain (California, US) |
| DIK | Wild-derived laboratory strain (Keshet, Israel) |
| DGA | Wild-derived laboratory strain (Adjaria, Georgia) |
| OR1400m105 | Inbred lab |
| BXD24 | Inbred lab |
| (CAST/EiJxC3H/HeJ)F1 | Inbred lab |
| BXD1 | Inbred lab |
| OR873f102 | Inbred lab |
| SB/LeJ | Inbred lab |
| NU/J | Inbred lab |
| OR1325m106 | Inbred lab |
| BXD9 | Inbred lab |
| (C57BL/6JxAKR/J)F1 | Inbred lab |
| BXD69 | Inbred lab |
| CBA/J | Inbred lab |
| OR906m104 | Inbred lab |
| OR804f103 | Inbred lab |
| OR1325f102 | Inbred lab |
| YBR/EiJ | Inbred lab |
| (PWD/PhJxNZW/LacJ)F1 | Inbred lab |
| BXD68 | Inbred lab |
| OR906f102 | Inbred lab |
| OR804m105 | Inbred lab |
| MRL/MpJ | Inbred lab |

| | |
|---|---|
| STX/Le | Inbred lab |
| OR1305f101 | Inbred lab |
| (PWD/PhJxNOD/ShiJ)F1 | Inbred lab |
| BXD67 | Inbred lab |
| 129X1/SvJ | Inbred lab |
| OR978m21 | Inbred lab |
| OR672m106 | Inbred lab |
| MA/MyJ | Inbred lab |
| ST/bJ | Inbred lab |
| OR1262f101 | Inbred lab |
| (PWD/PhJxC3H/HeJ)F1 | Inbred lab |
| BALB/cJ | Inbred lab |
| OR873m106 | Inbred lab |
| OR656f14 | Inbred lab |
| EL/SuzSeyFrkJ | Inbred lab |
| SJL/Bm | Inbred lab |
| OR496f18 | Inbred lab |
| OR656m18 | Inbred lab |
| DLS/LeJ | Inbred lab |
| SEC/1ReJ | Inbred lab |
| (NZW/LacJxPWD/PhJ)F1 | Inbred lab |
| (WSB/EiJxPWK/PhJ)F1 | Inbred lab |
| OR1611m121 | Inbred lab |
| OR95f16 | Inbred lab |
| CHMU/LeJ | Inbred lab |
| C57BLKS/J | Inbred lab |
| YCA1 | Inbred lab |
| SEC/1GnLeJ | Inbred lab |
| (NZW/LacJxC57BL/6J)F1 | Inbred lab |
| BXD60 | Inbred lab |
| (WSB/EiJxPWK/PhJ) | Inbred lab |
| OR1305m105 | Inbred lab |
| OR95m20 | Inbred lab |
| CE/J | Inbred lab |
| (NODxC57BLKS)F1 | Inbred lab |
| SJL/J | Inbred lab |
| (WSB/EiJxCAST/EiJ)F1 | Inbred lab |
| (129S1/SvImJxCAST/EiJ)F1 | Inbred lab |
| OR88f16 | Inbred lab |
| BXD65 | Inbred lab |
| (NZW/LacJxWSB/EiJ)F1 | Inbred lab |
| BXD73 | Inbred lab |

| | |
|---|---|
| BXD5 | Inbred lab |
| C3HeB/FeJ | Inbred lab |
| SH1/LeJ | Inbred lab |
| PN/nBSwUmabJ | Inbred lab |
| (CAST/EiJxNZW/LacJ)F1 | Inbred lab |
| BXD2 | Inbred lab |
| SEA/GnJ | Inbred lab |
| NZM2410/J | Inbred lab |
| C9 | Inbred lab |
| NOD/ShiLtJ | Inbred lab |
| (C57BL/6JxNZW/LacJ)F1 | Inbred lab |
| BXD49 | Inbred lab |
| (PWK/PhJxNOD/ShiJ)F1 | Inbred lab |
| (PWK/PhJx129S1/SvImJ)F1 | Inbred lab |
| ALS/LtJ | Inbred lab |
| C8 | Inbred lab |
| NOD.NON-Thy1(N13F21) | Inbred lab |
| RIIIS/J | Inbred lab |
| (C57BL/6JxNOD/ShiJ)F1 | Inbred lab |
| NZW/LacJ | Inbred lab |
| (PWK/PhJx129S1/SvImJ)F1 | Inbred lab |
| C7 | Inbred lab |
| (C57BL/6JxBALB/cJ)F1 | Inbred lab |
| NZO/HILtJ | Inbred lab |
| BXD44 | Inbred lab |
| (PWK/PhJxCAST/EiJ)F1 | Inbred lab |
| (PWK/PhJx129S1/SvImJ)F1 | Inbred lab |
| C6 | Inbred lab |
| PL/J | Inbred lab |
| LT/SvEiJ | Inbred lab |
| NOD/ShiLtJ | Inbred lab |
| BXD43 | Inbred lab |
| (PWK/PhJxC57BL/6J)F1 | Inbred lab |
| (129S1/SvImJIxPWK/PhJ)F1 | Inbred lab |
| C5 | Inbred lab |
| NZL/LtJ | Inbred lab |
| DBA/2HaSmnJ | Inbred lab |
| (AKR/JxC57BL/6J)F1 | Inbred lab |
| BXD42 | Inbred lab |
| OR1109m20 | Inbred lab |
| (PWK/PhJxC57BL/6J)F1 | Inbred lab |
| (129S1/SvImJIxPWK/PhJ)F1 | Inbred lab |

| | |
|---|---|
| C4 | Inbred lab |
| LP/J | Inbred lab |
| DBA/2DeJ | Inbred lab |
| BXD40 | Inbred lab |
| OR1109f19 | Inbred lab |
| (PWK/PhJxA/J)F1 | Inbred lab |
| (129S1/SvImJIxPWK/PhJ)F1 | Inbred lab |
| LG/J | Inbred lab |
| DBA/1LacJ | Inbred lab |
| BXD39 | Inbred lab |
| OR294f18 | Inbred lab |
| (PWK/PhJxA/J)F1 | Inbred lab |
| (129S1/SvImJIxPWK/PhJ)F1 | Inbred lab |
| JE/LeJ | Inbred lab |
| SEG1 | Inbred lab |
| BXD34 | Inbred lab |
| (NOD/ShiJxPWK/PhJ)F1 | Inbred lab |
| C58/J | Inbred lab |
| C57BL/10ScSnJ | Inbred lab |
| NONcNZO5/LtJ | Inbred lab |
| (DBA/2JxC57BL/6J)F1 | Inbred lab |
| (WSB/EiJxCAST/EiJ)F1 | Inbred lab |
| (CAST/EiJx129S1/SvImJ)F1 | Inbred lab |
| OR88m19 | Inbred lab |
| BUB/BnJ | Inbred lab |
| NONcNZO10/LtJ | Inbred lab |
| (BALB/cJxC57BL/6J)F1 | Inbred lab |
| (PWK/PhJxNOD/ShiJ)F1 | Inbred lab |
| (PWK/PhJx129S1/SvImJ)F1 | Inbred lab |
| ATEB/LeJ | Inbred lab |
| C57BLKS/J | Inbred lab |
| BDP/J | Inbred lab |
| BXD103MK88 | Inbred lab |
| BXD29 | Inbred lab |
| (CAST/EiJxPWK/PhJ)F1 | Inbred lab |
| DDY/JclSidSeyFrkJ | Inbred lab |
| RHJ/LeJ | Inbred lab |
| P/J | Inbred lab |
| C57BL/10J | Inbred lab |
| AEJ/GnRk | Inbred lab |
| BXD96 | Inbred lab |
| BXD28 | Inbred lab |

| | |
|---|---|
| (CAST/EiJxNOD/ShiJ)F1 | Inbred lab |
| DBA/2J | Inbred lab |
| HPG/BmJ | Inbred lab |
| A/WySnJ | Inbred lab |
| BXD21 | Inbred lab |
| (CAST/EiJxNOD/ShiJ)F1 | Inbred lab |
| DBA/1J | Inbred lab |
| C57BL/6J | Inbred lab |
| A/HeJ | Inbred lab |
| BXD20 | Inbred lab |
| (CAST/EiJxC57BL/6J)F1 | Inbred lab |
| BPN/3J | Inbred lab |
| C57BL/6J | Inbred lab |
| 129T2/SvEmsJ | Inbred lab |
| BXD16 | Inbred lab |
| (CAST/EiJxC57BL/6J)F1 | Inbred lab |
| BPH/2J | Inbred lab |
| C57BL/6J-Chr9/ForeJ | Inbred lab |
| 129P3/J | Inbred lab |
| (CAST/EiJxA/J)F1 | Inbred lab |
| CBA/CaJ | Inbred lab |
| AEJ/GnLeJ | Inbred lab |
| XBS1 | Inbred lab |
| 129P1/ReJ | Inbred lab |
| (C57B6/JxDBA/2J)F1 | Inbred lab |
| BPL/1J | Inbred lab |
| (CAST/EiJxA/J)F1 | Inbred lab |
| OR1048m20 | Inbred lab |
| I/LnJ | Inbred lab |
| C57BL/6J-Chr19/ForeJ | Inbred lab |
| C57BL/6J-Chr18/ForeJ | Inbred lab |
| ILS | Inbred lab |
| BXD13 | Inbred lab |
| (CAST/EiJx129S1/SvImJ)F1 | Inbred lab |
| OR1048f18 | Inbred lab |
| C57BL/6NJ | Inbred lab |
| C57BL/6J-Chr10.3/ForeJ | Inbred lab |
| OR1587m104 | Inbred lab |
| ISS | Inbred lab |
| C57BL/6NCr | Inbred lab |
| BXD86 | Inbred lab |
| BXD84 | Inbred lab |

| | |
|---|---|
| BXD12 | Inbred lab |
| (C57BL/6JxPWK/PhJ)F1 | Inbred lab |
| C57L/J | Inbred lab |
| C57BL/10ScNJ | Inbred lab |
| BXD32 | Inbred lab |
| (CAST/EiJxWSB/EiJ)F1 | Inbred lab |
| KK/HIJ | Inbred lab |
| OR1018f102 | Inbred lab |
| RF/J | Inbred lab |
| C57BR/cdJ | Inbred lab |
| BXSB/MpJ | Inbred lab |
| BXD31 | Inbred lab |
| (CAST/EiJxPWK/PhJ)F1 | Inbred lab |
| FVB/NJ | Inbred lab |
| RSV/LeJ | Inbred lab |
| C57BL/6NJ | Inbred lab |
| C57BL/6J-Chr12/ForeJ | Inbred lab |
| OR151f102 | Inbred lab |
| IBWSR2 | Inbred lab |
| C57BL/6NCr | Inbred lab |
| (C57BL/6JxPWK/PhJ)F1 | Inbred lab |
| OR496m20 | Inbred lab |
| ZRDCT Rax+/ChUmdJ | Inbred lab |
| C57BL/6NJ | Inbred lab |
| DDK/Pas | Inbred lab |
| ICOLD2 | Inbred lab |
| C57BL/6NCrl | Inbred lab |
| (C57BL/6JxCAST/EiJ)F1 | Inbred lab |
| 129S6 | Inbred lab |
| TSJ/LeJ | Inbred lab |
| C57BL/6NJ | Inbred lab |
| C57BL/6J | Inbred lab |
| C57BL/6J-Chr11.1/ForeJ | Inbred lab |
| IHOT2 | Inbred lab |
| BXD48 | Inbred lab |
| TKDU/DnJ | Inbred lab |
| (129X1/SvJxCAST/EiJ)F1 | Inbred lab |
| C57BL/6J | Inbred lab |
| C3H/HeJ | Inbred lab |
| IHOT1 | Inbred lab |
| BXD45 | Inbred lab |
| OR672f102 | Inbred lab |

| | |
|---|---|
| TALLYHO/JngJ | Inbred lab |
| ALR/LtJ | Inbred lab |
| C57BL/6J | Inbred lab |
| BTBRT+tf/J | Inbred lab |
| BXD102 | Inbred lab |
| SWR/J | Inbred lab |
| C57BL/6J | Inbred lab |
| BALB/cByJ | Inbred lab |
| SSL/LeJ | Inbred lab |
| C57BL/6J | Inbred lab |
| NOR/LtJ | Inbred lab |
| ZRU1 | Inbred lab |
| BXD100 | Inbred lab |
| AKR/J | Inbred lab |
| BXD75 | Inbred lab |
| ICR/HaJ | Inbred lab |
| C57BL/6J | Inbred lab |
| OR447f19 | Inbred lab |
| NON/ShiLtJ | Inbred lab |
| BXD99 | Inbred lab |
| BXD18 | Inbred lab |
| BXD74 | Inbred lab |
| A/J | Inbred lab |
| SM/J | Inbred lab |
| C57BL/6J | Inbred lab |
| OR294m21 | Inbred lab |
| NZB/BINJ | Inbred lab |
| (WSB/EiJxNZW/LacJ)F1 | Inbred lab |
| BXD33 | Inbred lab |
| C57BL/6NJ | Inbred lab |
| OR1587f101 | Inbred lab |
| PN/nBSwUmabJ | Inbred lab |
| IBWSP2 | Inbred lab |
| C57BL/6NTac | Inbred lab |
| BXD61 | Inbred lab |
| BXD83 | Inbred lab |
| BXD8 | Inbred lab |
| (A/JxPWK/PhJ)F1 | Inbred lab |
| OR1244m19 | Inbred lab |
| OR1005m105 | Inbred lab |
| 129S1SvlmJ | Inbred lab |
| C57BL/6NJ | Inbred lab |

| | |
|---|---|
| C57BL/6J-Chr14/ForeJ | Inbred lab |
| OR151m105 | Inbred lab |
| C57BL/6NTac | Inbred lab |
| BXD6 | Inbred lab |
| (A/JxPWK/PhJ)F1 | Inbred lab |
| OR615m104 | Inbred lab |
| OR1005f102 | Inbred lab |

Table S1: **Empirical sample names and their corresponding type and origin (City, Province, Country). Origin was reported for the wild caught and wild-derived laboratory strains.**

| **Chromosome** | **Introgressed tract** | **Tract left-endpoint** | **Tract right-endpoint** | **Length (in bp)** |
|---|---|---|---|---|
| 7 | 1 | 102119720 | 104379248 | 2259528 |
| 7 | 2 | 106602021 | 107313081 | 711060 |
| 7 | 3 | 123047532 | 133421080 | 10373548 |
| 15 | 1 | 58688840 | 60258289 | 1569449 |
| 15 | 2 | 75714436 | 78398411 | 2683975 |
| 17 | 1 | 8600043 | 9226619 | 626576 |
| 17 | 2 | 9777661 | 10274843 | 497182 |
| 17 | 3 | 12067936 | 12404484 | 336548 |
| 17 | 4 | 25048414 | 25930707 | 882293 |
| 17 | 5 | 26256724 | 26854062 | 597338 |
| 17 | 6 | 28835086 | 31400633 | 2565547 |

Table S2: **Genomic coordinates for the introgressed tracts in the empirical study.** The left and right endpoints, and length of each introgressed tract are shown for chromosome 7, 15, and 17.

# References

[1] DeLong, E.R., DeLong, D.M., Clarke-Pearson, D.L.: Comparing the areas under two or more correlated receiver operating characteristic curves: a nonparametric approach. Biometrics, 837–845 (1988)

[2] Benjamini, Y., Hochberg, Y.: Controlling the false discovery rate: A practical and powerful approach to multiple testing. Journal of the Royal Statistical Society Series B (Methodological) **57**(1), 289–300 (1995)
